# An Efficient Method for the In Vitro Production of Azol(in)e-Based Cyclic Peptides**

**DOI:** 10.1002/anie.201408082

**Published:** 2014-10-21

**Authors:** Wael E Houssen, Andrew F Bent, Andrew R McEwan, Nathalie Pieiller, Jioji Tabudravu, Jesko Koehnke, Greg Mann, Rosemary I Adaba, Louise Thomas, Usama W Hawas, Huanting Liu, Ulrich Schwarz-Linek, Margaret C M Smith, James H Naismith, Marcel Jaspars

**Affiliations:** Marine Biodiscovery Centre, Department of Chemistry, University of AberdeenMeston Walk, Aberdeen AB24 3UE (UK); Institute of Medical Sciences, University of AberdeenAberdeen AB25 2ZD (UK); Pharmacognosy Department, Faculty of Pharmacy, Mansoura UniversityMansoura 35516 (Egypt); Biomedical Sciences Research Complex, University of St AndrewsNorth Haugh, St Andrews, Fife KY16 9ST (UK); Department of Biology, University of YorkWentworth Way, York YO10 5DD (UK); Marine Chemistry Department, Faculty of Marine Sciences, King Abdulaziz UniversityJeddah 21589 (Saudi Arabia)

**Keywords:** cyanobactins, cyclic peptides, biosynthesis, patellamides, ribosomal peptides

## Abstract

Heterocycle-containing cyclic peptides are promising scaffolds for the pharmaceutical industry but their chemical synthesis is very challenging. A new universal method has been devised to prepare these compounds by using a set of engineered marine-derived enzymes and substrates obtained from a family of ribosomally produced and post-translationally modified peptides called the cyanobactins. The substrate precursor peptide is engineered to have a non-native protease cleavage site that can be rapidly cleaved. The other enzymes used are heterocyclases that convert Cys or Cys/Ser/Thr into their corresponding azolines. A macrocycle is formed using a macrocyclase enzyme, followed by oxidation of the azolines to azoles with a specific oxidase. The work is exemplified by the production of 17 macrocycles containing 6–9 residues representing 11 out of the 20 canonical amino acids.

Macrocyclic peptides show high target affinity, bioavailability, and stability and thus have enjoyed considerable use as therapeutics.^1^ The conformational constraints on macrocyclic peptides imposed by the incorporation of heterocycles have been suggested to contribute to higher receptor affinity by reducing the entropic penalty paid for immobilization.^2^ The testing and development of such constrained macrocyclic compounds is hindered by the technical difficulties and the high cost of their chemical synthesis on a useful scale.^3^ Several cyanobacteria have been found to produce diverse bioactive azole-containing cyclic peptides, the cyanobactins, with the most well-known being the patellamides. These conformationally constrained peptides are made by post-translational tailoring of ribosomal peptides.^4^ Using enzymes and substrates from the patellamide, trunkamide, aestuaramide, microcyclamide, and tenuecyclamide biosynthetic pathways, we present a robust scalable in vitro route for the production of azoline-containing cyclic peptides (Scheme 1). The thiazoline-containing products can be further treated with oxidases derived from the *Cyanothece* PCC 7425 or *Arthrospira platensis* to obtain thiazoles which are less prone to spontaneous epimerization at the adjacent stereocenters.^5^

**Scheme 1:**
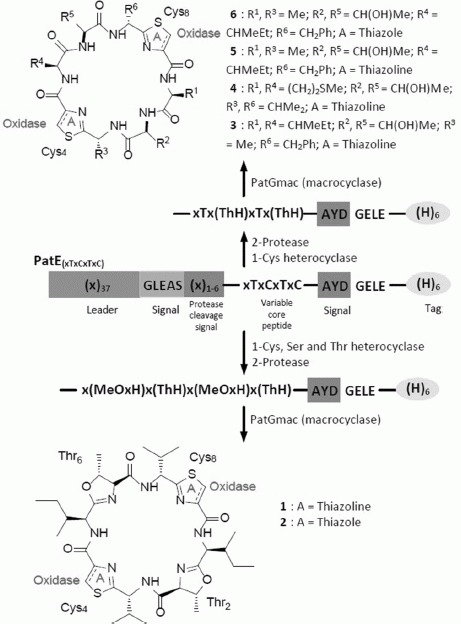
A schematic representation of the in vitro biosynthesis of azol(in)e-based cyclic peptides. The substrate PatE is first processed with either TruD/LynD (which convert Cys into thiazoline) or PatD/MicD/TenD (which convert Cys, Ser, and Thr into thiazoline, oxazoline, and methyl oxazoline, respectively). The leader sequence of the purified and processed substrate is then cleaved off with a suitable protease. The cleaved core peptide is purified and cyclized with PatG_mac_. Thiazoline rings can be oxidized to thiazoles with the oxidases from *Cyanothece* (Thc_oxi_) or *A. platensis* (Ap_oxi_).

The route is flexible as we can use either the cysteine- (or selenocysteine-) specific heterocyclases, TruD (*Prochloron* sp.) or LynD (*Lyngbya* sp.) which only slowly process Ser or Thr,^6^, ^7^ or the heterocyclases PatD (*Prochloron* sp.), MicD (*M. aeruginosa*), or TenD (*N. spongiaeforme var. tenue*) that readily process Thr, Ser, Se-Cys, and Cys (Scheme 2, Figure S1). Although we have used PatG_mac_^8^ (from the patellamide pathway) as the macrocyclase, it will be straightforward to introduce macrocyclases from other organisms which are predicted to macrocyclize different sized rings.

**Scheme 2:**
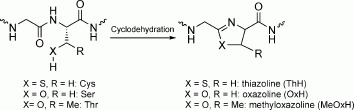
Heterocyclization reaction of cysteine, serine, or threonine residues.

We use a modified substrate PatE_(core sequence)_ with a single core peptide sequence and a histidine tag at the C-terminus to aid the purification. PatE natural peptides may contain one or more core sequences; each is flanked at the N-terminus with a protease cleavage signal and at the C-terminus with PatG_mac_ signal. We can overexpress such peptides to a high level in *E. coli* (100–200 mg L^−1^) and with our protocol solubilize the protein from inclusion bodies.6a

We have shown elsewhere that the N-terminal leader sequence can be shortened and still retain the essential recognition determinants for the heterocyclase.6a After heterocyclization the thiazoline- (or oxazoline-) containing substrate must be processed prior to macrocyclization involving cleavage of the N-terminal leader. Analysis of chemical reactivity implies that epimerization at Cα precedes oxidation but follows heterocyclization; however, the exact sequence of the chemical reactions in vivo remains unknown. To provide insight into the epimerization reaction, a heterocyclized (two thiazolines) perdeuterated PatE_(ITACITFC)_ (^2^H-PatE_(ITACITFC)_) sample was prepared and an ^1^H NMR spectrum was immediately recorded. In the event of a spontaneous epimerization reaction, exchange of peptide Cα deuterium with hydrogen from the solvent would be observed as an increase in the NMR signal. No increase in the signal was observed immediately after heterocyclization or on the same sample following incubation at pH 9.0 for seven days (Figure S2). This suggests epimerization, if it is spontaneous, will occur in the macrocycle as previously predicted.^9^

PatA, the cognate protease which cleaves after the GLEAS motif is extremely slow in vitro and thus not suitable for the production of milligram quantities of material or a large number of variant patellamides.^10^ It has been shown that it is possible to insert at least six residues between GLEAS and the core peptide without affecting the heterocyclization reaction.6a,b The introduction of specific protease sites is a well-established tool in protein chemistry.^11^ We, therefore, have developed a number of solutions to replace PatA by inserting other protease sites between the core and the GLEAS motif. Insertion of a single Lys residue allows trypsin to cut very rapidly (Figure S3) but of course is not suitable for core sequences containing Lys or Arg unless the subsequent residue is Pro (or possibly a heterocyclized residue). Tobacco etch virus (TEV) protease is very efficient but as its recognition site is ENLYFQ↑G it changes the first residue of the core to Gly (TEV protease tolerates other residues except proline) (Figure S4).^12^ GluC selectively cleaves peptide bonds C-terminal to Glu, but in our studies it was poor in terms of speed and yield.

We used PCR Based Mutagenesis with the In-Fusion HD Cloning System (Clontech Laboratories, USA) to generate a series of PatE substrates. Alternatively, we have developed new vectors encoding the PatE peptide, with TEV protease or trypsin N-terminal cleavage sites, where we can incorporate short oligonucleotides, which cover only the core peptide sequence, into the vector by simple annealing and thus facilitate the design of the final products (Figures S5 and S6).

Oxidation of the thiazoline rings in **1** to thiazoles was achieved using *Cyanothece* oxidase (Thc_oxi_) in the presence of FMN cofactor (Figure S7). With this protein we did not observe activity on the heterocycle-containing linear peptides. In contrast, we could demonstrate oxidation of both linear PatE and macrocyclic product with the highly homologous enzyme Ap_oxi,_ from *A. platensis* (Figures S8 and S9). The oxidation of the heterocycles in linear substrates has previously been characterized in the thiazole/oxazole-modified microcins (TOMMS) pathway.^13^ The dehydrogenation reaction could alternatively be carried out at a much slower rate by reaction with excess MnO_2_ in dichloromethane for three days at 28 °C. Circular dichroism measurements showed that the stereochemistry of the final product obtained by chemical oxidation was identical to that of the natural product ascidiacyclamide (Figure S10). This indicates that epimerization had occurred spontaneously at the stereocenters adjacent to the thiazolines as previously predicted.^9^ It remains unclear whether and to what extent the different oxidases are sensitive to the stereochemical context of the heterocycle (has epimerization occurred and has the macrocycle formed). Final products are purified from PatG_mac_ reaction mixtures using SPE followed by HPLC.

The method is scalable and 1–2 mg of highly pure final product can be obtained from each 100 mL macrocyclization reaction containing 100 μm of cleaved and processed PatE. Using this approach, we have successfully synthesized, isolated, and characterized a small library of azol(in)e-containing cyclic peptides of six to nine amino acids (compounds **1**–**17**; Table 1). These compounds have been generated from variable core sequences with 11 out of the 20 canonical amino acids. NMR spectra and LCMS data were recorded for compounds **1**, **3**, **4**, and **7** while LCMS and MSMS analyses were used to confirm the identity of the other compounds (NMR data are listed in Tables S1–S4, NMR and LCMS spectra are shown in Figures S11–S43). Compound **1** is the reduced form of ascidiacyclamide and was previously isolated, for the first time, by our group from a specimen of *Lissoclinum patella*. Figure S12 a shows the stacked ^1^H NMR spectra of the natural and biosynthetic materials.

**Table 1:** List of compounds generated by in vitro biosynthesis.

	Core sequence	Amino acid sequence in the modified cyclic product
**1**	ITVCITVC^[a]^	I(MeOxH)V(ThH)I(MeOxH)V(ThH)
**2**	ITVCITVC^[b]^	I(MeOxH)V(Thz)I(MeOxH)V(Thz)
**3**	ITACITFC^[c]^	ITA(ThH)ITF(ThH)
**4**	MTVCMTVC^[c]^	MTV(ThH)MTV(ThH)
**5**	ATACITFC^[c]^	ATA(ThH)ITF(ThH)
**6**	ATACITFC^[d]^	ATA(Thz)ITF(Thz)
**7**	IMACIMAC^[c]^	IMA(ThH)IMA(ThH)
**8**	ITACITAC^[c]^	ITA(ThH)ITA(ThH)
**9**	ITACISFC^[c]^	ITA(ThH)ISF(ThH)
**10**	GITACICVC^[c]^	ITA(ThH)I(ThH)V(ThH)
**11**	VCVCVC^[c]^	V(ThH)V(ThH)V(ThH)
**12**	ITMCITMC^[c]^	ITM(ThH)ITM(ThH)
**13**	IFTVCICVC^[c]^	IFTV(ThH)I(ThH)V(ThH)
**14**	ITACITYC^[c]^	ITA(ThH)ITY(ThH)
**15**	ITACITYC^[a]^	I(MeOxH)A(ThH)I(MeOxH)Y(ThH)
**16**	IDACIDFC^[c]^	IDA(ThH)IDF(ThH)
**17**	IACIMAC^[c]^	IA(ThH)IMA(ThH)

Core sequences are processed with [a] PatD, trypsin, and PatG_mac_; [b] PatD, trypsin, PatG_mac_, and Thc_oxi_; [c] TruD, trypsin, and PatG_mac_; [d] TruD, trypsin, PatG_mac_,and Ap_oxi_.

A successful in vivo approach^14^ to produce highly modified cyanobactins has been reported and shown to be capable of producing a cyanobactin with a nonnatural amino acid. Our in vitro approach has some key advantages over the in vivo approach. The in vitro approach allows the same precursor peptide to give different final products (for example by processing one portion with PatD and another portion with TruD or by using oxidase or not). This avoids the complex protein-engineering approach that would be required in vivo. The in vitro process is quicker as it uses more active proteases and tunes the conditions for each reaction, rather than accepting a single compromise. The in vitro approach is essential for the production of compounds that can inhibit the growth of the in vivo host (antibacterial). Finally the in vitro approach allows facile real-time monitoring and intervention. On the other hand, the in vivo approach has its merits by being much cheaper and less labor intensive (no need for enzyme purification).

From a purely synthetic viewpoint, macrocyclic peptides are challenging as macrocyclization is often low-yielding requiring reactions to be carried out with low concentrations in large reaction volumes to favor macrocyclization over oligomerization.^15^ Biosynthetic alternatives include sortase-mediated ligation, but this requires an LPXTG motif at the C-terminus and oligo-G at the N terminus, which are incorporated in the final cyclic peptide.^16^ Similarly protein splicing requires the synthesis of a linear peptide containing intein, signals for which are again incorporated in the final peptide unless additional steps are carried out. This method is often inefficient and >30 % of sequences cannot be cyclized. Problems associated with the chemical synthesis of thiazolines and oxazolines include the likely racemization at the labile α-carbon adjacent to the thiazoline, the low yield, and the side reactions.^17^

In summary, our approach will open up the synthesis of large numbers of cyanobactin variants in biologically useful quantities. This will in turn revolutionize their application in biology and in the longer term therapeutic discovery, which is currently stalled because no useful or generally applicable routes exist to such molecules.

## Supporting Information

Supporting information for this article (including experimental details) is available on the WWW under http://dx.doi.org/10.1002/anie.201408082.

As a service to our authors and readers, this journal provides supporting information supplied by the authors. Such materials are peer reviewed and may be re-organized for online delivery, but are not copy-edited or typeset. Technical support issues arising from supporting information (other than missing files) should be addressed to the authors.

miscellaneous_information
