# Supplementary material for: An Efficient Method for the In Vitro Production of Azol(in)e-Based Cyclic Peptides
Source: Angew Chem Int Ed Engl. 2014 Oct 21;53(51):14171–4. doi: 10.1002/anie.201408082 (PMC4282754; doi:10.1002/anie.201408082)
Supplement: Supplementary file 1 — miscellaneous_information [file anie0053-14171-sd1.pdf]

Angewandte Chemie
Eine Zeitschrift der Gesellschaft Deutscher Chemiker

Supporting Information

© Wiley-VCH 2014

69451 Weinheim, Germany

# An Efficient Method for the In Vitro Production of Azol(in)e-Based Cyclic Peptides**

*Wael E. Houssen, Andrew F. Bent, Andrew R. McEwan, Nathalie Pieiller, Jioji Tabudravu, Jesko Koehnke, Greg Mann, Rosemary I. Adaba, Louise Thomas, Usama W. Hawas, Huanting Liu, Ulrich Schwarz-Linek, Margaret C. M. Smith, James H. Naismith,* and Marcel Jaspars**

anie_201408082_sm_miscellaneous_information.pdf

# Supporting information

## Experimental Data

### Cloning, expression and purification of enzymes

*a- Heterocyclases*

Codon-optimized full length PatD and TruD were cloned into the pJexpress 411 plasmid (DNA2.0 Inc., USA) with an N-terminal $His_6$-tag, with TruD containing an additional Tobacco Etch Virus (TEV) protease cleavage site before the tag. Both enzymes are expressed in *Escherichia coli* BL21 (DE3) cells grown on auto-induction medium (Terrific broth base containing trace elements) for 48 h at 20 °C. Cells are harvested by centrifugation at 4,000 x g, 4 °C for 15 min. Pellets are re-suspended in 500 mM NaCl, 20 mM Tris pH 8.0, 20 mM imidazole and 3 mM BME and supplemented with 0.4 mg DNAse $g^{-1}$ wet cells (Sigma) and complete protease inhibitor tablets (EDTA-free, Roche). Cells are lyzed by passage through a cell disruptor at 30 kPSI or by sonication and the lysates are cleared by centrifugation at 40,000 x g, 4 °C for 45 min followed by filtration through 0.4 µm membrane filter. Cleared lysates are applied to a Ni-sepharose FF column (GE Healthcare) pre-washed with lysis buffer and the protein eluted with 250 mM Imidazole. The $His_6$-tag of TruD is removed by addition of 1 mg TEV protease per 10 mg TruD incubated at room temperature for 2 hours and the cleaved protein isolated by passage through a second Ni-sepharose FF column. (Note: TruD still functions efficiently if $His_6$-tag is attached). Both enzymes are then loaded on to a Superdex 200 gel filtration column (GE Healthcare), pre-equilibrated and run in 150 mM NaCl, 10 mM HEPES pH 7.4, 1 mM TCEP. Peak fractions were pooled and the proteins concentrated to 100 µM for use in *in vitro* reactions.

Codon optimised DNA sequence encoding PatD
ATGCATCATCACCATCACCACTTGGTGCCGCGCGGTTCTCAGCCGACCGCGTTGCAGATCAAAC
CGCATTTTCATGTGGAGATCATCGAGCCGAAACAGGTGTACCTGCTGGGCGAGCAGGGCAATC
ACGCCCTGACGGGTCAACTGTATTGCCAAATCCTGCCGTTTCTGAATGGCGAGTATACCCGCGA
GCAAATCGTGGAGAAGTTGGACGGCCAAGTCCCGGAAGAGTATATCGACTTTGTCCTGTCCCGC
CTGGTCGAGAAGGGCTACCTGACCGAGGTGGCGCCTGAGTTGAGCCTGGAAGTTGCAGCGTTC
TGGAGCGAATTGGGCATCGCACCTTCCGTGGTGGCGGAAGGTCTGAAACAACCGGTTACCGTT
ACCACCGCTGGCAAGGGCATTCGCGAGGGTATCGTCGCAAATCTGGCAGCCGCGCTGGAAGAG
GCGGGCATCCAGGTCAGCGATCCGAAAGCCCCGAAAGCACCGAAGGCAGGCGATTCGACGGC
ACAACTGCAAGTGGTGCTGACCGACGATTACCTGCAGCCGGAATTGGCGGCCATCAACAAAGA
GGCTCTGGAGCGTCAGCAGCCGTGGCTGCTGGTTAAACCGGTGGGCAGCATCCTGTGGCTGGGT
CCGCTGTTTGTCCCTGGTGAAACGGGTTGTTGGCACTGCCTGGCCCAACGCCTGCGCGGCAACC
GTGAGGTTGAGGCGTCTGTTCTGCAACAGAAGCGTGCGTTGCAAGAGCGTAACGGTCAGAATA
AGAACGGTGCAGTTAGCTGTCTGCCGACGGCTCGTGCGACGCTGCCGAGCACCCTGCAGACCG
GCCTGCAGTGGGCAGCGACCGAGATCGCCAAGTGGATGGTTAAACGTCACCTGAACGCGATTG
CTCCGGGTACGGCGCGTTTCCCGACTCTGGCGGGTAAGATTTTCACCTTCAACCAGACGACGCT
GGAACTGAAAGCCCATCCGCTGAGCCGTCGTCCGCAATGTCCGACGTGCGGTGACCAGGAGAT
TCTGCAGCGTCGTGGTTTTGAACCGCTGAAGCTGGAATCTCGCCCGAAACACTTTACGTCCGAT
GGTGGTCACCGTGCCACCACCCCGGAACAGACCGTGCAGAAATACCAGCACCTGATTGGTCCG
ATTACTGGTGTGGTGACCGAGCTGGTGCGTATTAGCGATCCGGCAAACCCGCTGGTGCATACGT
ATCGTGCGGGCCACAGCTTTGGTAGCAGCGCAGGCAGCCTGCGTGGCTTGCGTAATACCTTGCG
TTACAAGTCTAGCGGTAAGGGCAAGACTGACAGCCAGTCCCGTGCAAGCGGTCTGTGTGAAGC
GATCGAGCGCTACAGCGGCATCTTTCTGGGTGACGAACCGCGTAAACGCGCGACCCTGGCTGA
ACTGGGTGATCTGGCGATTCATCCGGAGCAATGCCTGCATTTCTCTGACCGCCAATACGACAAT
CGCGATGCGTTGAACGCGGAAGGCAGCGCGGCAGCGTACCGTTGGATTCCGCACCGTTTTGCG
GCGAGCCAGGCGATTGATTGGACGCCGCTGTGGAGCCTGACGGAACAGAAGCACAAGTACGTT
CCAACGGCTATTTGCTACTACAACTACCTGCTGCCACCGGCGGACCGTTTCTGCAAAGCGGACA
GCAACGGTAACGCGGCTGGTAACTCGTTGGAAGAAGCCATCCTGCAGGGTTTCATGGAACTGG
TTGAGCGTGATTCTGTGGCCCTGTGGTGGTATAACCGCCTGCGTCGCCCGGAGGTCGAGTTGAG
CAGCTTCGAAGAACCGTACTTCCTGCAACTGCAACAATTCTACCGCAGCCAAAATCGCGAATTG

TGGGTTCTGGACCTGACTGCTGACTTGGGTATTCCGGCATTTGCCGGCCTGAGCCGTCGTACGG
TCGGTTCCAGCGAGCGTGTCTCCATTGGTTTCGGTGCCCACTTGGACCCGAAGATCGCCATTCT
GCGCGCTCTGACGGAGGTTAGCCAAGTCGGTCTGGAGCTGGATAAGGTCCCGGATGAGAAGCT
GGACGGTGAAAGCAAAGATTGGATGCTGGAAGTTACCCTGGAAACGCATCCGTGCCTGGCTCC
TGATCCATCTCAGCCGCGTAAGACCGCGAATGACTATCCAAAGCGTTGGAGCGATGACATTTAC
ACCGATGTGATGGCGTGTGTTGAAATGGCAAAAGTGGCAGGTCTGGAGACTCTGGTCCTGGAT
CAGACCCGTCCGGACATTGGTCTGAATGTTGTTAAAGTCATGATCCCAGGTATGCGCACCTTTT
GGAGCCGCTATGGTCCTGGCCGTCTGTATGACGTTCCGGTGCAACTGGGTTGGCTGAAAGAGCC
ACTGGCAGAGGCGGAGATGAACCCGACCAATATCCCATTCTAA

Codon optimised DNA sequence encoding TruD
ATGCATCACCACCACCATCACGAAAACCTGTACTTCCAAGGCCAGCCGACTGCACTGCAGATCA
AACCGCATTTTCACGTGGAGATTATTGAACCGAAACAGGTGTACCTGTTGGGTGAGCAGGGCA
ATCACGCGCTGACGGGCCAGCTGTATTGCCAGATCTTGCCGTTTCTGAATGGTGAATACACCCG
CGAGCAGATTGTGGAGAAGTTGGATGGTCAGGTGCCGGAGGAGTACATTGATTTCGTTCTGAG
CCGTCTGGTTGAAAAGGGCTATTTGACCGAAGTGGCTCCGGAGTTGAGCCTGGAGGTCGCGGC
GTTCTGGTCCGAGCTGGGTATTGCGCCGAGCGTCGTGGCCGAGGGCCTGAAACAACCGGTTACC
GTCACCACGGCGGGTAAGGGTATCCGCGAGGGTATCGTCGCCAATCTGGCGGCAGCGTTGGAA
GAAGCGGGCATCCAGGTGAGCGACCCGCGTGATCCGAAAGCACCGAAGGCAGGCGACTCTACC
GCACAGCTGCAAGTCGTGCTGACGGACGATTATCTGCAGCCGGAGCTGGCGGCGATTAACAAA
GAAGCTCTGGAGCGTCAGCAACCGTGGTTGCTGGTTAAGCCGGTCGGTAGCATCTTGTGGCTGG
GTCCGCTGTTCGTTCCGGGTGAAACGGGTTGCTGGCACTGTCTGGCCCAGCGCCTGCAAGGCAA
TCGTGAGGTGGAAGCTAGCGTCCTGCAGCAGAAACGCGCACTGCAGGAGCGCAACGGTCAAAA
CAAGAACGGTGCCGTTAGCTGCCTGCCGACCGCGCGTGCGACCCTGCCTAGCACCTTGCAGACT
GGCCTGCAGTGGGCTGCTACCGAGATTGCGAAATGGATGGTGAAACGTCATCTGAATGCAATT
GCTCCGGGCACGGCACGTTTCCCGACGCTGGCGGGCAAAATCTTTACGTTTAATCAGACCACGC
TGGAACTGAAGGCGCACCCGCTGTCTCGTCGCCCACAATGTCCGACTTGCGGTGACCGTGAGAC
TCTGCAGCGCCGTGGTTTCGAGCCTCTGAAGCTGGAAAGCCGCCCTAAACACTTTACGAGCGAT
GGTGGTCACCGTGCGATGACCCCGGAACAAACGGTTCAGAAGTACCAGCATCTGATTGGTCCA
ATTACGGGCGTTGTCACCGAACTGGTGCGTATTAGCGATCCGGCGAACCCGTTGGTCCATACCT
ACCGTGCAGGCCACTCCTTCGGTAGCGCGACCAGCCTGCGCGGTCTGCGTAACGTCTTGCGTCA
CAAGTCTAGCGGTAAAGGTAAGACCGATTCCCAAAGCCGCGCAAGCGGCCTGTGTGAGGCCAT
TGAACGTTATAGCGGCATTTTTCAAGGTGACGAGCCGCGTAAGCGCGCCACCCTGGCGGAGCT
GGGCGACCTGGCTATCCATCCGGAACAATGCTTGCATTTCAGCGATCGTCAATATGATAACCGC
GAAAGCAGCAACGAACGTGCAACCGTCACGCATGACTGGATCCCGCAACGTTTTGACGCCTCT
AAGGCACACGACTGGACCCCGGTTTGGAGCCTGACGGAGCAAACCCACAAGTACCTGCCGACG
GCGCTGTGTTATTACCGTTACCCGTTTCCGCCAGAGCATCGTTTCTGCCGTTCCGACTCGAACGG
TAACGCAGCGGGTAATACCCTGGAGGAAGCGATCCTGCAGGGTTTCATGGAACTGGTTGAGCG
CGATAGCGTGTGTCTGTGGTGGTATAATCGCGTGAGCCGTCCGGCCGTGGACCTGTCTAGCTTC
GATGAACCTTACTTCCTGCAACTGCAACAGTTTTACCAGACCCAGAACCGTGATTTGTGGGTGC
TGGATCTGACCGCGGACTTGGGTATTCCAGCTTTCGTGGGCGTTTCCAATCGTAAAGCAGGCAG
CAGCGAGCGCATTATCTTGGGCTTTGGTGCCCACCTGGATCCGACCGTTGCCATCCTGCGTGCG
CTGACCGAGGTGAATCAAATTGGCCTGGAGCTGGACAAAGTTAGCGACGAATCCCTGAAAAAT
GACGCGACGGATTGGCTGGTTAATGCGACTCTGGCCGCAAGCCCGTATCTGGTGGCGGATGCTA
GCCAACCTCTGAAAACCGCAAAGGATTACCCGCGTCGTTGGAGCGACGACATCTATACCGAC
GTCATGACCTGCGTTGAGATCGCTAAACAAGCAGGCCTGGAAACGCTGGTCCTGGACCAGACC
CGTCCGGACATCGGCCTGAACGTCGTTAAGGTTATCGTTCCAGGTATGCGCTTTTGGAGCCGCT
TTGGTAGCGGTCGTCTGTACGACGTGCCGGTCAAACTGGGTTGGCGTGAGCAACCGCTGGCGG
AAGCACAAATGAACCCGACGCCGATGCCATTCTAA

*b- Macrocyclase*

PatG$_{mac}$ (PatG residues 492–851) was cloned from genomic DNA (*Prochloron* sp.) into the pHISTEV vector and expressed in *Escherichia coli* BL21 (DE3) grown on auto-induction medium (Terrific broth base containing trace elements) for 48 h at 20 °C. Cells are harvested by centrifugation at 4,000 x g, 4 °C, for 15 min and re-suspended in lysis buffer (500 mM NaCl, 20 mM Tris, pH 8.0, 20 mM imidazole and 3 mM

BME with the addition of complete EDTA-free protease inhibitor tablets (Roche) and 0.4 mg DNase (Sigma) $g^{-1}$ wet cells. Cells are lysed by passage through a cell disruptor at 30 kPSI or by sonication, and the lysate is cleared by centrifugation at 40,000 x g, 4 °C for 45 min followed by filtration through 0.4 µm membrane filter. Cleared lysate is applied to a Ni-sepharose FF column (GE Healthcare) column prewashed with lysis buffer, and protein was eluted with 250 mM imidazole. The protein is then passed over Superdex 75 (GE Healthcare) in 150 mM NaCl, 10 mM HEPES pH 7.4, 1 mM TCEP and concentrated to 1 mM.

*c- Oxidases*

The gene encoding the thiazoline oxidase ($Thc_{oxi}$) was amplified from the *Cyanothece* PCC 7425 *g*DNA and cloned into the pJexpress 401 plasmid (DNA2.0 Inc., USA) with an N-terminal $His_6$-tag and TEV protease cleavage site. The enzyme was expressed in *Escherichia coli* BL21 (DE3) cells grown on auto-induction medium (Terrific broth base containing trace elements) for 48 h at 20 °C. Cells are harvested by centrifugation at 4,000 x g, 4 °C for 15 min. Pellets are re-suspended in 500 mM NaCl, 20 mM Tris pH 8.0, 20 mM imidazole, 3 mM BME and supplemented with 0.4 mg DNAse $g^{-1}$ wet cells (Sigma) and complete protease inhibitor tablets (EDTA-free, Roche). Cells are lyzed by sonication and the lysates are cleared by centrifugation at 40,000 x g, 4 °C for 45 min followed by filtration through 0.4 µm membrane filter. Cleared lysates are applied to a Ni-sepharose FF column (GE Healthcare) pre-washed with lysis buffer and the protein eluted with 250 mM Imidazole. The enzyme was then loaded on to a Superdex 75 gel filtration column (GE Healthcare) in 500 mM NaCl, 20 mM Tris pH 8.0, 1 mM TCEP and concentrated to 0.5 mM.

$Ap_{oxi}$ (ApG residues 2–481) was cloned into the pJexpress 401 plasmid (DNA2.0 Inc., USA) with an *N*-terminal $His_6$-tag and TEV protease cleavage site. The enzyme was expressed in *Escherichia coli* BL21 (DE3) cells grown on auto-induction medium (Terrific broth base containing trace elements) supplemented with 50 µM riboflavin for 48 h at 20 °C. Cells are harvested by centrifugation at 4,000 x g, 4 °C for 15 min. Pellets are re-suspended in 500 mM NaCl, 20 mM Tris pH 8.0, 20 mM imidazole, 3 mM BME and 50 µM FMN and supplemented with 0.4 mg DNAse $g^{-1}$ wet cells (Sigma) and complete protease inhibitor tablets (EDTA-free, Roche). Cells are lyzed by passage through a cell disruptor at 30 kPSI and the lysates are cleared by centrifugation at 40,000 x g, 4 °C for 45 min followed by filtration through 0.4 µm membrane filter. Cleared lysates are applied to a Ni-sepharose FF column (GE Healthcare) pre-washed with lysis buffer and the protein eluted with 250 mM Imidazole. The $His_6$-tag is removed by addition of 1 mg TEV protease per 10 mg $Ap_{oxi}$ incubated at room temperature for 2 hours and the cleaved protein isolated by passage through a second Ni-sepharose FF column. The enzyme was then loaded on to a Superdex 200 gel filtration column (GE Healthcare) in 150 mM NaCl, 10 mM HEPES pH 7.4, 1 mM TCEP and concentrated to 0.5 mM.

*d- Precursor peptides*

Variants of PatE, each encoding only one core peptide instead of two tandem patellamide core peptides, were cloned with a C-terminal $His_6$-tag into pBMS23 for easier analysis of processed products. To enable more efficient N-terminal cleavage, additional residues were in some cases added directly before the core peptide to allow for cleavage by either trypsin (K/R) or TEV (ENLYFQ).
We used PCR Based Mutagenesis with the In-Fusion HD Cloning System to generate diversity. We also have developed a simple cloning strategy where we can incorporate short oligonucleotides, which cover only the core peptide sequence, into vectors (Fig. S5 & S6) by simple annealing. A detailed description of this strategy is as below:

**Construction of pETCHISPatE_K_var1 (Fig. S5)**

To generate pETCHISPatE_K_var1, a PCR amplification was carried out using a forward oligo (5'GCATCACTTTTTGCGCATATGATGGTG 3' *Nde*I site underline) and a reverse oligo (5'TTTAGATGCTTCCAAACCAG 3') with construct pBMS23CHISPatE(K) as a template. Proofreading Pfu DNA polymerase was used in the amplification to generate a blunt end PCR product. The specific amplified DNA products were separated in electrophoresis and extracted from the gel. The linearized pETCHISPatE_K _var1 was obtained by purifying the DNA fragments after *Nde*I digestion.

**Construction of pETCHISpatE_K_var2 (Fig. S6)**

To generate pETCHISPatE_K _var2, a PCR amplification was carried out using Pfu DNA polymerase with a forward oligo (5'GCATCACTTTTTGCGCATATGATGGTG 3' *Hind* III site underline), a reverse oligo (5' CCATCATATGCGCAAAAAGTGATGCAA GCAGAGGCCTTAGATGC 3', *Nde*I and *Stu*I underline) and construct pBMS23CHISPatE(K) as a template. The amplified DNA fragments were separated in electrophoresis, extracted from the gel and digested by *Nde*I restriction enzyme. pETCHISPatE_K _proV2 was generated by ligation the *Nde*I-digested DNA fragments and then transformed into DH5α. The transformed cells were cultured and the pETCHISPatE_K _proV2 DNA was prepared. The linearized pETCHISPatE_K _var2 was obtained by purifying the DNA fragment after *Stu* I/*Nde*I-digestion of pETCHISPatE_K _proV2 DNA.

**Application of pETCHISPatE_K _var1 and var2**

1. Forward oligos with the designed codons marked with x and three fixed bases (GCA) at its 3' end were synthesised (Fig. S5).
2. Complementary oligo of the forward strand plus TA at its 5' end was synthesised (for library generation bases marked x in the oligos can be degenerated).
3. The two oligos were mixed in an equal molar ratio and the mixture was heated at 95ºC for 5 minutes.
4. The mixture was slowly cooled to room temperature.
5. The annealed oligos were phosphorylated with T4 PNK followed by T4 PNK inactivation.
6. The annealed double strand oligo was ligated into the pETCHISPatE_TEV_var.
7. The ligation mixture was transformed to DH5α and plasmids were extracted from overnight cultures prepared from single colonies.

NB: Residue variations of pETCHISPatE_K _var2 in position 1 are limited to Val, Ala, Asp, Glu and Gly.

**Generation of pETCHISPatE_TEV_var**

To generate pETCHISPatE_TEV_var, PCR amplification was carried out using a forward oligo (5'CTGGCCGGCATATGATGGTGAACTC3' *Nde* I site underline) and a reverse oligo (5'CTGAAAATACAGGTTTTCAG3') with construct pBMS23CHISPatE (TEV) as a template. Proofreading Pfu DNA polymerase was used in the amplification to generate a blunt end PCR product. The specific amplified DNA products were separated in electrophoresis and extracted from the gel. The linearized pETCHISPatE_TEV was obtained by purifying the DNA fragments after *Nde* I digestion.

**Application of pETCHISPatE_TEV _var**

1. Forward oligos with the designed codons marked with X and three fixed bases GCA at its 3' end were synthesised (Fig. S6).
2. Complementary oligo of the forward strand plus TA at its 5' end was synthesised (for library generation bases marked x in the oligos can be degenerated).
3. The two oligos were mixed in an equal molar ratio and the mixture was heated at 95ºC for 5 minutes.
4. The mixture was slowly cooled to room temperature.
5. The annealed oligos were phosphorylated with T4 PNK followed by T4 PNK inactivation.
6. The annealed double strand oligo was ligated into the pETCHISPatE_TEV_var.
7. The ligation mixture was transformed to DH5α and plasmids were extracted from overnight cultures prepared from single colonies.

PatE protein is expressed in BL21(DE3) cells grown on auto-induction medium (Terrific broth base containing trace elements) at 37 °C overnight. Cells are harvested by centrifugation at 4,000 x g, 20 °C, for 15 min and re-suspended in 8 M urea, 500 mM NaCl, 20 mM Tris pH 8.0, 20 mM imidazole and 3 mM BME. Cells are lyzed by sonication, and the lysate is cleared by centrifugation at 40,000 x g, 20 °C for 45 min followed by filtration through 5, 0.8 and 0.4 µm membrane filters respectively. Cleared lysate is applied to a Ni-sepharose FF column (GE Healthcare) column prewashed with lysis buffer, and protein was eluted with 250 mM imidazole. DDT is added to the eluted PatE to a final concentration of 10 mM and the solution is incubated at room temperature for 3 hours. PatE is further purified and separated from protein aggregates

by size-exclusion chromatography (Superdex 75, GE Healthcare) in 150 mM NaCl, 10 mM HEPES pH 7.4, 1 mM TCEP and concentrated to 1 mM.

For $^{2}$H-PatE$_{(ITACITFC)}$ a freshly transformed *E. coli* BL21 DE3 colony of PatE$_{(ITACITFC)}$ in the pBMS23 plasmid was conditioned for growth in deuterated media through sequential growth on LB-agar plates made in increasing $D_2O$ concentration up to 100 %. The adapted cells were streaked onto an M9 minimal media-agar [F. C. Neidhardt, P. L. Bloch, D. F. Smith, *J. Bacteriol.* **1974**, *119*, 736-747.] plate made in 100 % $D_2O$, and a subsequent colony was used to inoculate a 25 mL 100 % $D_2O$/M9 minimal media culture and grown at 37 °C until $OD_{600}$ = 1. The 25 mL culture was diluted into 500 mL fresh 100 % $D_2O$/M9 minimal media and grown at 37 °C until $OD_{600}$ = 0.6. Cells were induced with 1 mM IPTG, and grown at 30 °C for 30 h. Cells were harvested and purified as for native PatE with the final protein dialyzed into PBS buffer prior to NMR experiments.

## *In vitro* reactions

### *a- Heterocyclization reaction*

Heterocyclization reaction contains 100 µM PatE, 0.5 µM TruD /PatD, 5 mM ATP pH 7, 5 mM $MgCl_2$, 150 mM NaCl, 10 mM HEPES, pH 7.4, 1 mM TCEP. Reactions are incubated at 37 °C with shaking at 200 rpm for 24 h when using TruD and 48 h for PatD. In some cases the PatE shows a degree of precipitation. In these instances the peptide is recovered from the precipitate by denaturation in 8M urea as above followed by Ni affinity chromatography and size-exclusion. Reactions are monitored by MALDI-MS. Processed PatE is purified on Superdex 75, GE Healthcare in 150 mM NaCl, 10 mM HEPES pH 7.4, 1 mM TCEP and concentrated.

### *b- Proteolytic cleavage*

Different proteases can be used e.g. trypsin or TEV protease depending on the PatE sequence created. There remains scope for further proteases. We use 4 µg of trypsin per 1 mg of purified processed PatE. The corresponding figure for TEV protease is 1 mg for each 10 mg of PatE. Reactions are incubated at 37 °C with shaking at 200 rpm for up to 4 hours. Reaction products are purified using Superdex 30, GE Healthcare in 150 mM NaCl, 20 mM Bicine pH 8.0. The purified product could be concentrated using on Phenomenex© Strata C18-E, 55µm, 70Å, 2g/12 mL Giga SPE tube cartridges. After loading the sample, a washing step with deionised water to get rid of buffer salts is carried out and this is followed by elution step with 5× column volume methanol and 5× column volume of acetonitrile. We also wash the column with 5× volume of 0.1% TFA in acetonitrile. Washings with water or acidified acetonitrile are tested separately by MS for any peptides but in our experience, peptides are eluted completely with the organic solvents.

### *c- Macrocyclization reaction*

Macrocyclization reactions contain 100 µM peptide, 5 % DMSO, 500 mM NaCl, 20 µM PatG$_{mac}$, and 20 mM bicine pH 8.0. Reactions are incubated at 37 °C with shaking at 200 rpm for 4 days and monitored by MS. Reaction volumes are concentrated on Phenomenex© Strata C18-E, 55µm, 70Å, 2g/12 mL Giga SPE tube cartridges following the above procedure. This is followed by final purification of the products using HPLC on C4 ACE column 10 x 250 mm, 5 µm and using a gradient of acetonitrile in water. Purification process is monitored using DAD at wavelengths of 210, 220, 230, 240 and 254 nm.

### *d- Macrocycle oxidation reaction*

**Chemical oxidation:** This was carried out using $MnO_2$ in dichloromethane for three days at 28 °C. The resulting mixture was subjected to silica gel and celite column chromatography followed by HPLC chromatography to yield the oxidized product.

**Thc$_{oxi}$ reaction:** Pure heterocycles-containing cyclic peptide was dissolved in DMSO and diluted to a final concentration of 20 µM cyclic peptide and 0.5% DMSO in a reaction mixture containing 50 µM FMN co-

factor, 10 µM $Thc_{oxi}$, 500 mM NaCl and 20 mM Tris pH 8.0. The reaction was incubated overnight at 37 °C with shaking at 200 rpm. The reaction product was analyzed by LC-ESIMS.

**$Ap_{oxi}$ reaction:** 200 µM of heterocycles-containing cyclic peptide was incubated overnight at 37 °C with 20 µM $Ap_{oxi}$ and 1 mM FMN co-factor. The reaction was analyzed by MALDI-MS.

*e- Linear PatE oxidation reaction*

**$Ap_{oxi}$ reaction:** Full length PatE (200 µM) was incubated overnight at 37 °C with 20 µM $Ap_{oxi}$ and 1 mM FMN co-factor. The reaction was purified on a Superdex 75, GE Healthcare in 150 mM NaCl, 10 mM HEPES pH 7.4, 1 mM TCEP and analyzed by MALDI-MS.

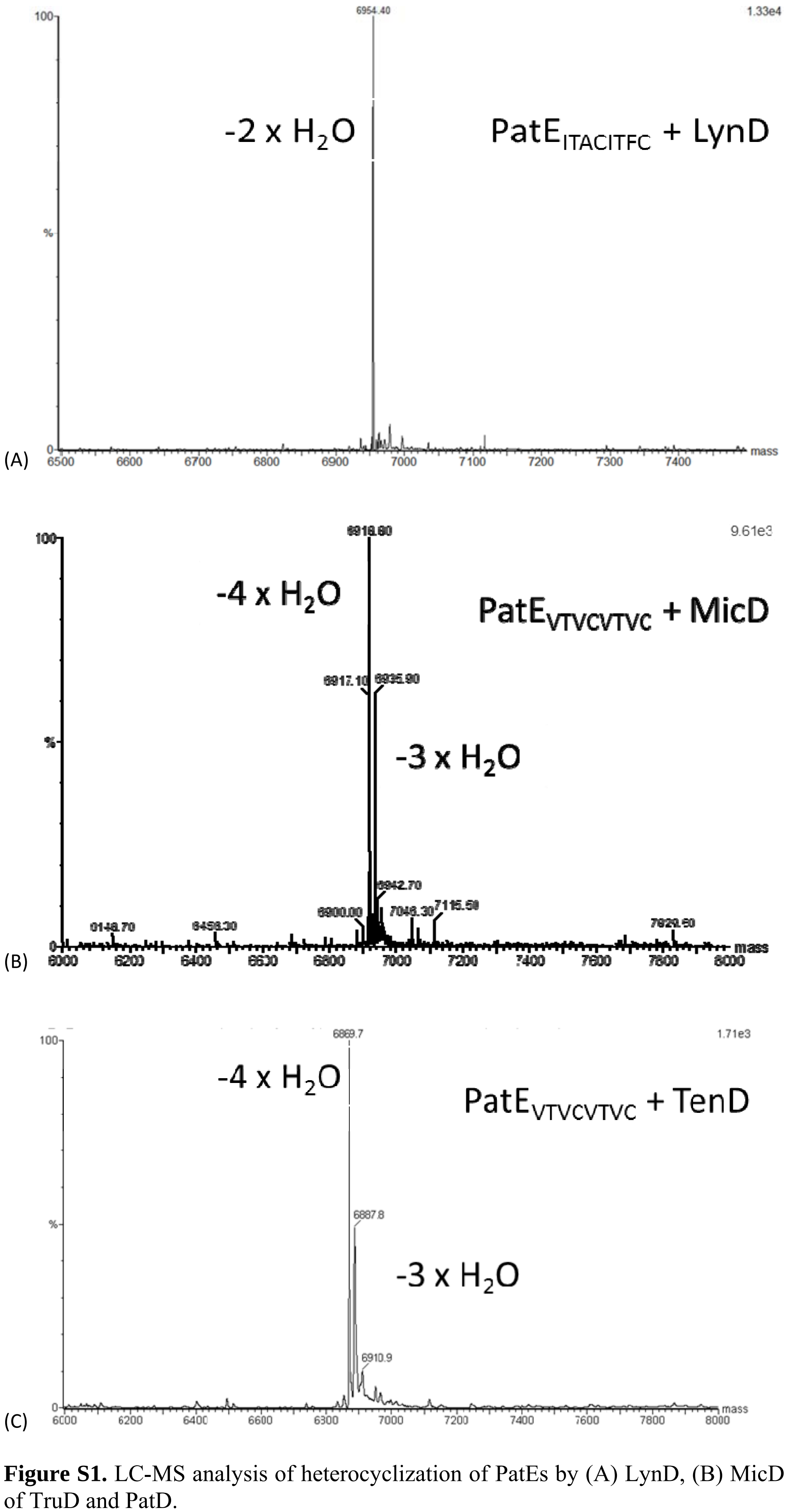


**Figure S1.** LC-MS analysis of heterocyclization of PatEs by (A) LynD, (B) MicD and (C) TenD; homologs of TruD and PatD.

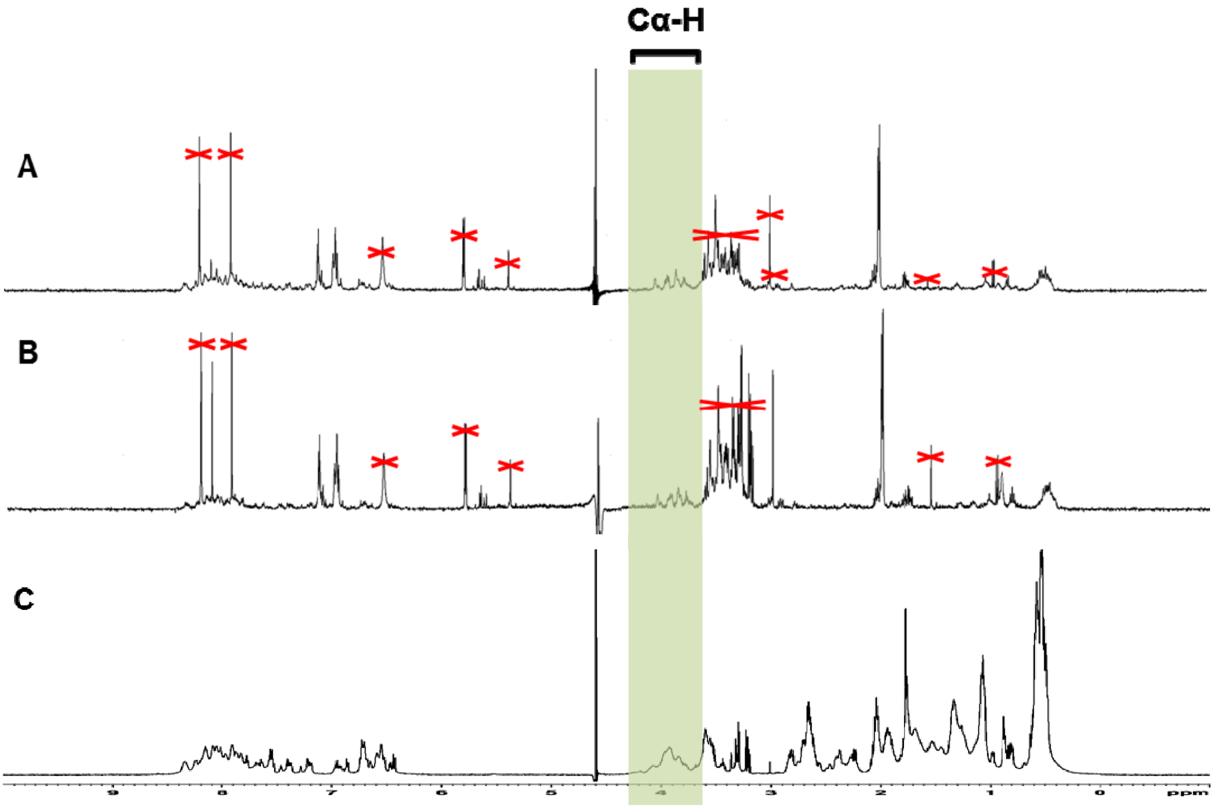


**Figure S2.** $^{1}H$ NMR of heterocyclized $^{2}$H-PatE$_{(ITACITFC)}$. **A)** $^{2}$H-PatE$_{(ITACITFC)}$ immediately after heterocyclization by TruD. **B)** $^{2}$H-PatE$_{(ITACITFC)}$ heterocyclized by TruD and incubated for 7 days at pH 9.0. **C)** Heterocyclized $^{2}$H-PatE$_{(ITACITFC)}$ for comparison. For each spectra peaks attributed to small molecules (ATP, TCEP and HEPES) retained after dialysis have been crossed out in red. The Cα region has been highlighted in green. No obvious change within the Cα region indicates epimerization is not chemically spontaneous on the linear peptide.

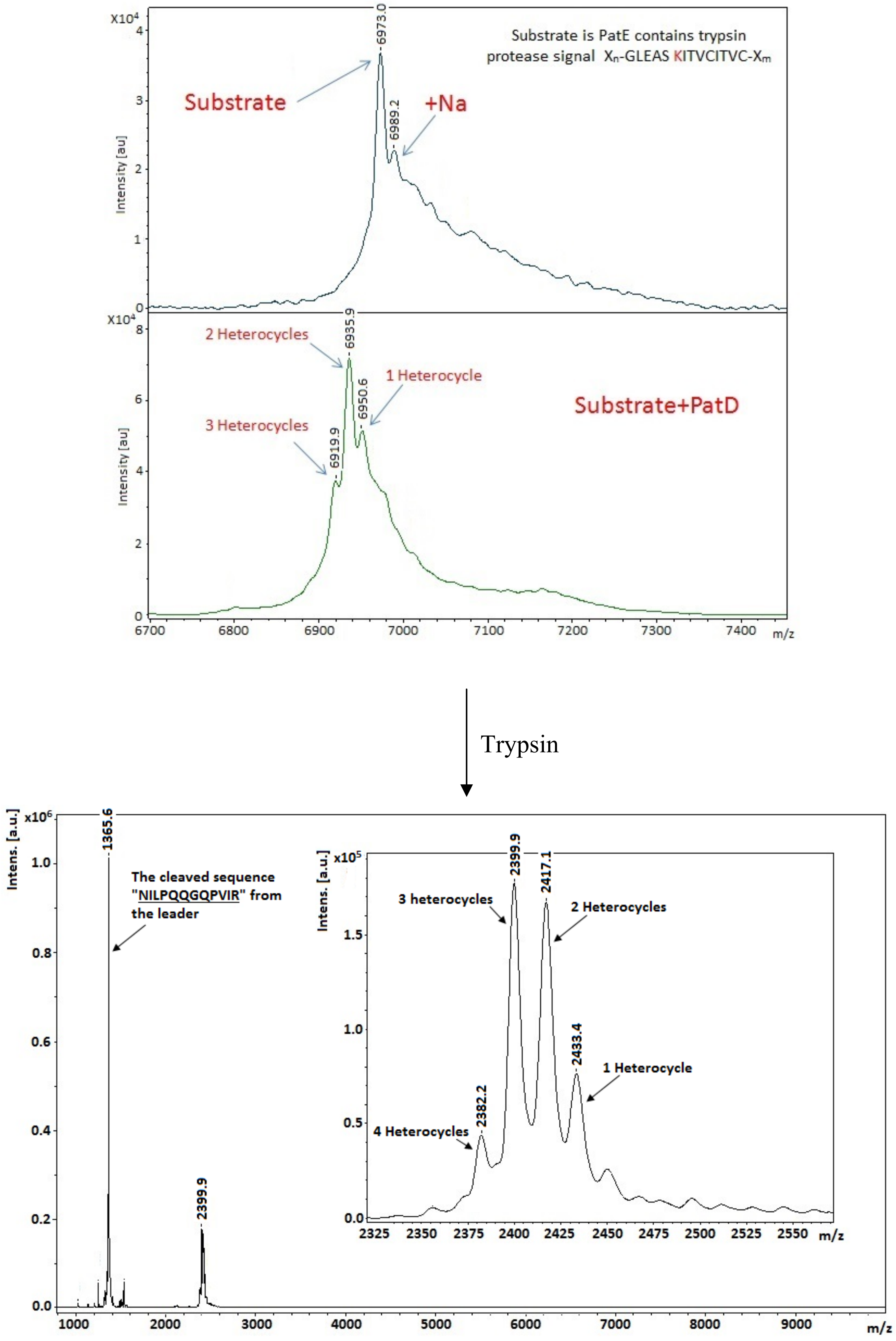


**Figure S3.** PatE substrate treated with PatD for 24 h after which the leader is cleaved by trypsin. Both reactions were monitored by MALDI. Formation of a single product containing 4 heterocycles needs longer incubation (48 h) with PatD.

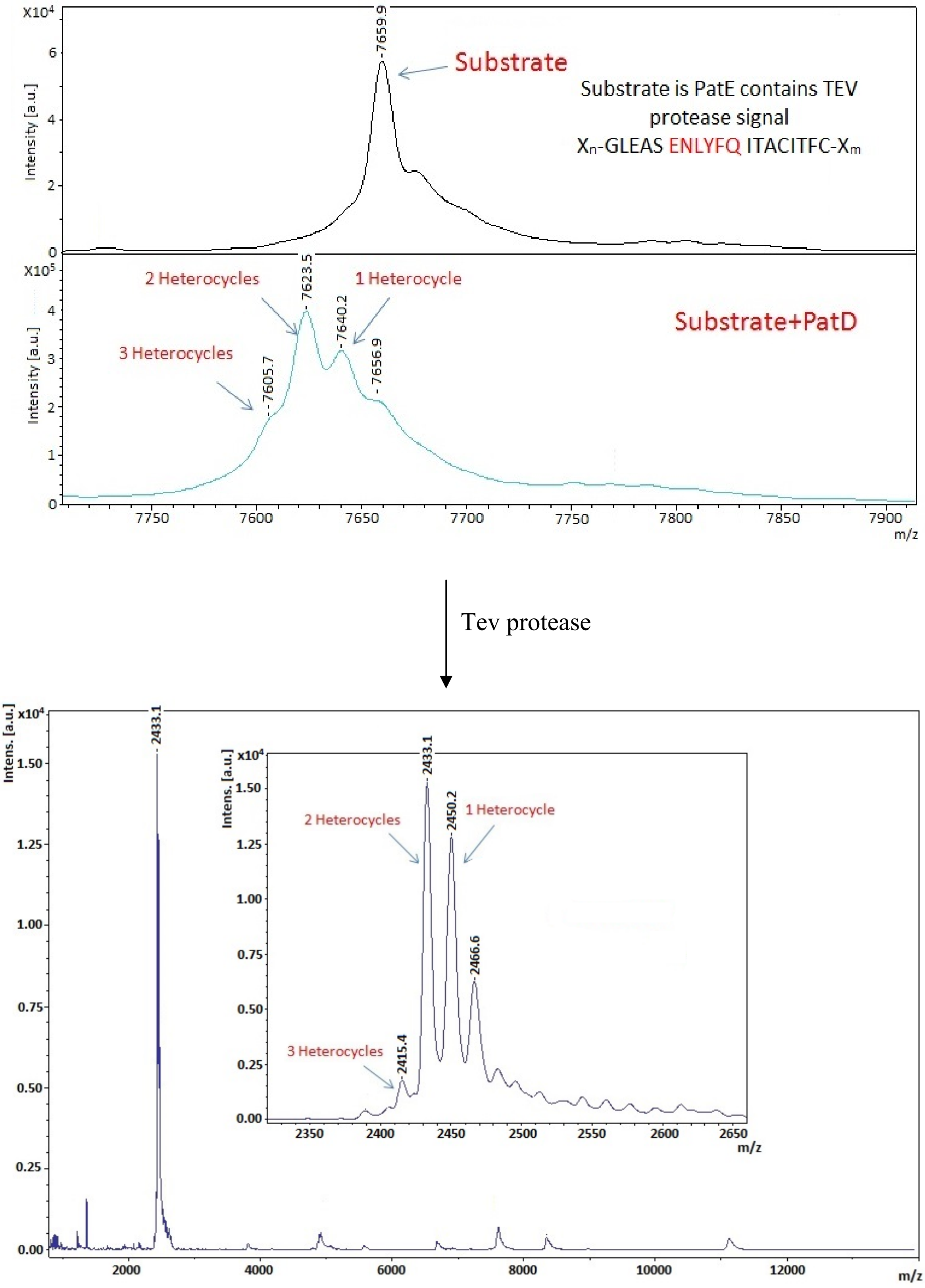


**Figure S4.** PatE substrate treated with PatD for 24 h after which the leader is cleaved by TEV protease. Both reactions were monitored by MALDI. Formation of a single product containing 4 heterocycles needs longer incubation (48 h) with PatD.

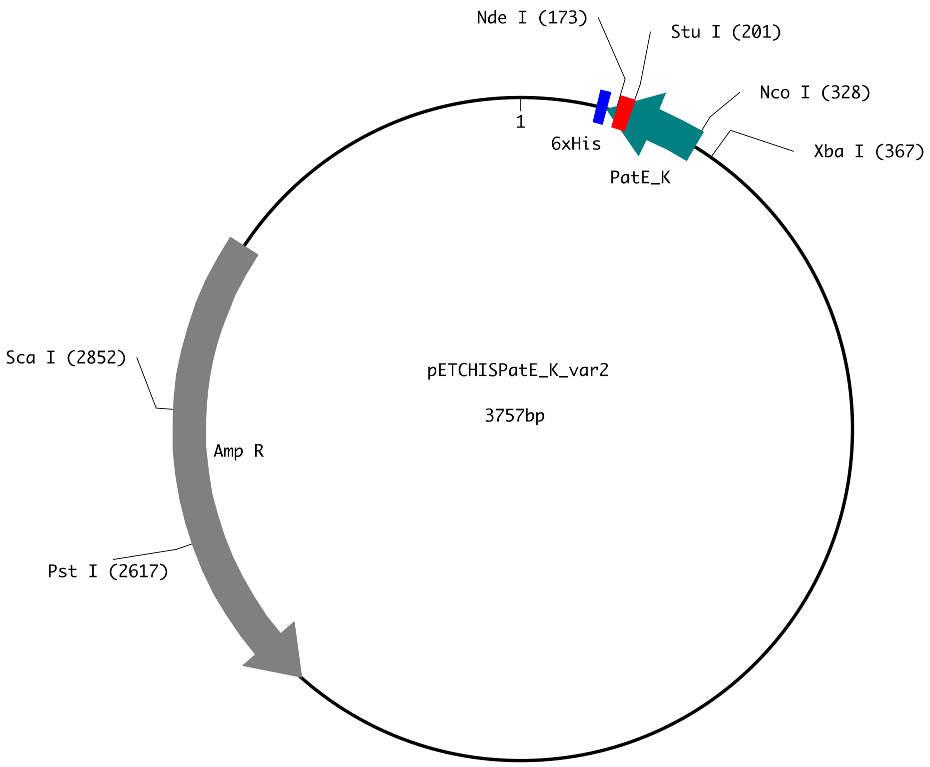


```
                        1    2    3    4    5    6    7    8
5'TTG GAA GCA TCT AAA xxx xxx xxx xxx xxx xxx xxx xxx GCA TAT GAT GGT GAA CTC3'
3'AAC CTT CGT AGA TTT xxx xxx xxx xxx xxx xxx xxx xxx CGT ATA CTA CCA CTT GAG5'
   L   E   A   S   K                                   A   Y   D   G   E   L
```

**Figure S5.** pETCHISPatE_K _var1 plasmid map. PatE gene was cloned into pBMS23CHIS using restriction enzyme *Nco* I/*Xho* I digestions, the constructed pBMS23CHISPatE (K) express PatE with 6x histidines at it C-terminus.

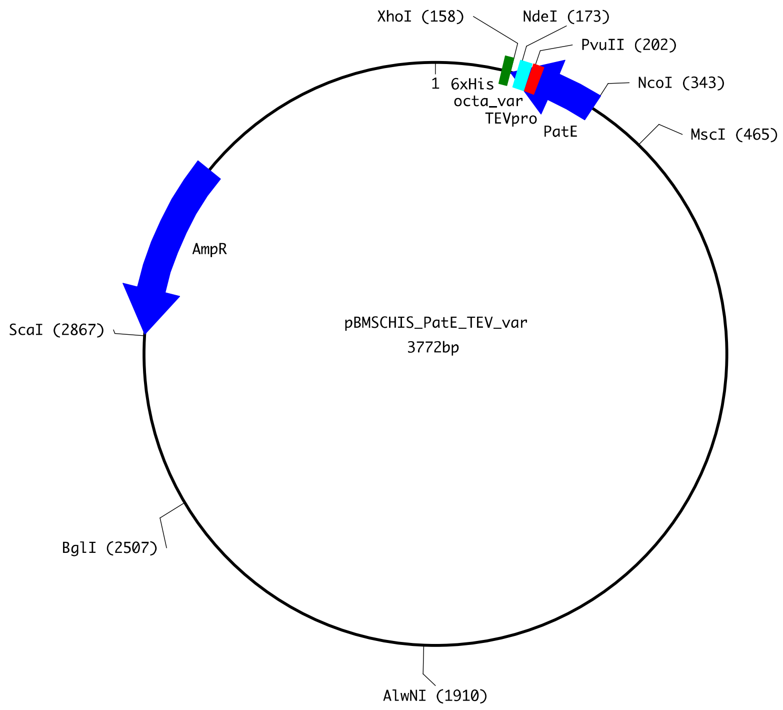


```
                          1    2    3    4    5    6    7    8
5'AAC CTG TAT TTT CAG xxx xxx xxx xxx xxx xxx xxx xxx GCA TAT GAT GGT GAA CTC3'
3'TTG GAC ATA AAA GTC xxx xxx xxx xxx xxx xxx xxx xxx CGT ATA CTA CCA CTT GAG5'
   N   L   Y   F   Q                                   A   Y   D   G   E   L
```

**Figure S6.** pETCHISPatE_TEV_var plasmid map. PatE gene was cloned into pBMSCHIS using restriction enzyme *Nco* I/*Xho* I digestions and a TEV protease site coding sequence was inserted upstream of the octa-residue region. The constructed pBMS23CHISPatE (TEV) express PatE with 6x histidines at it C-terminus and a TEV protease site immediately upstream of the core peptide residues.

a.)

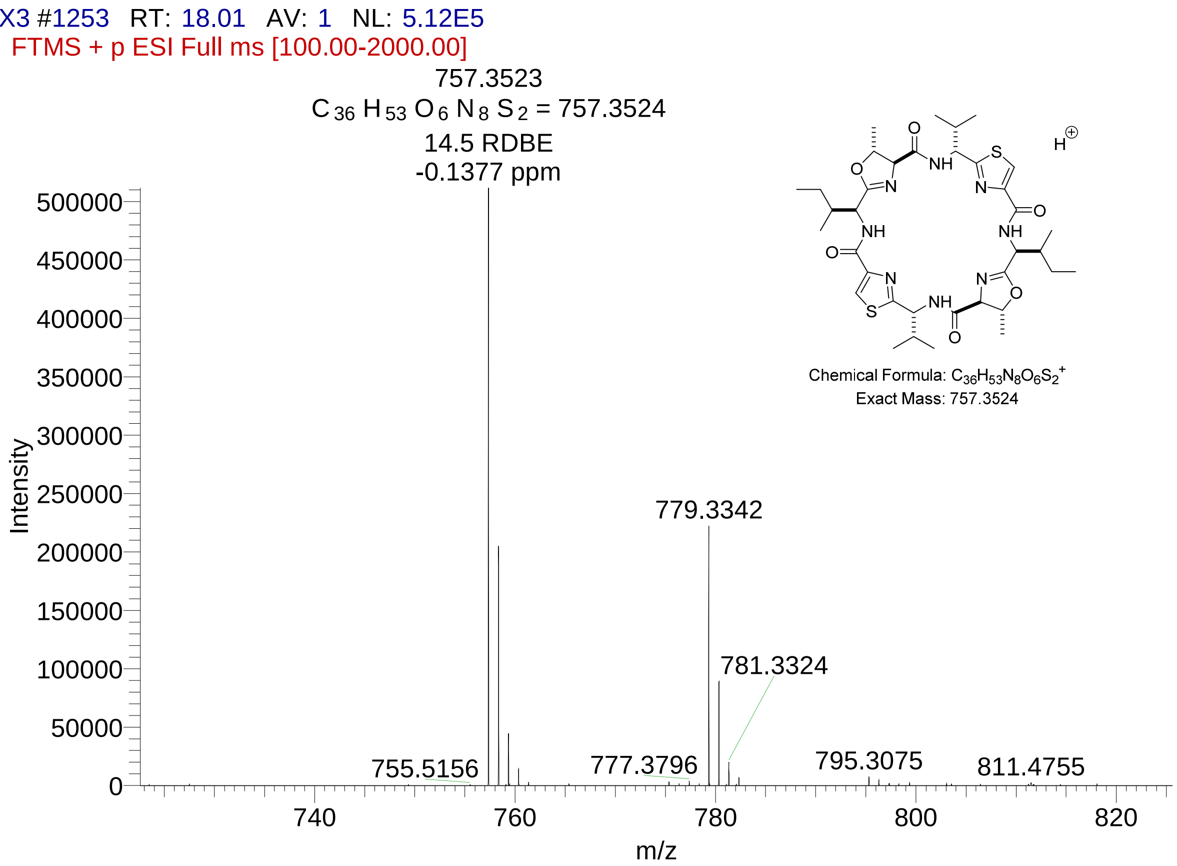


b.)

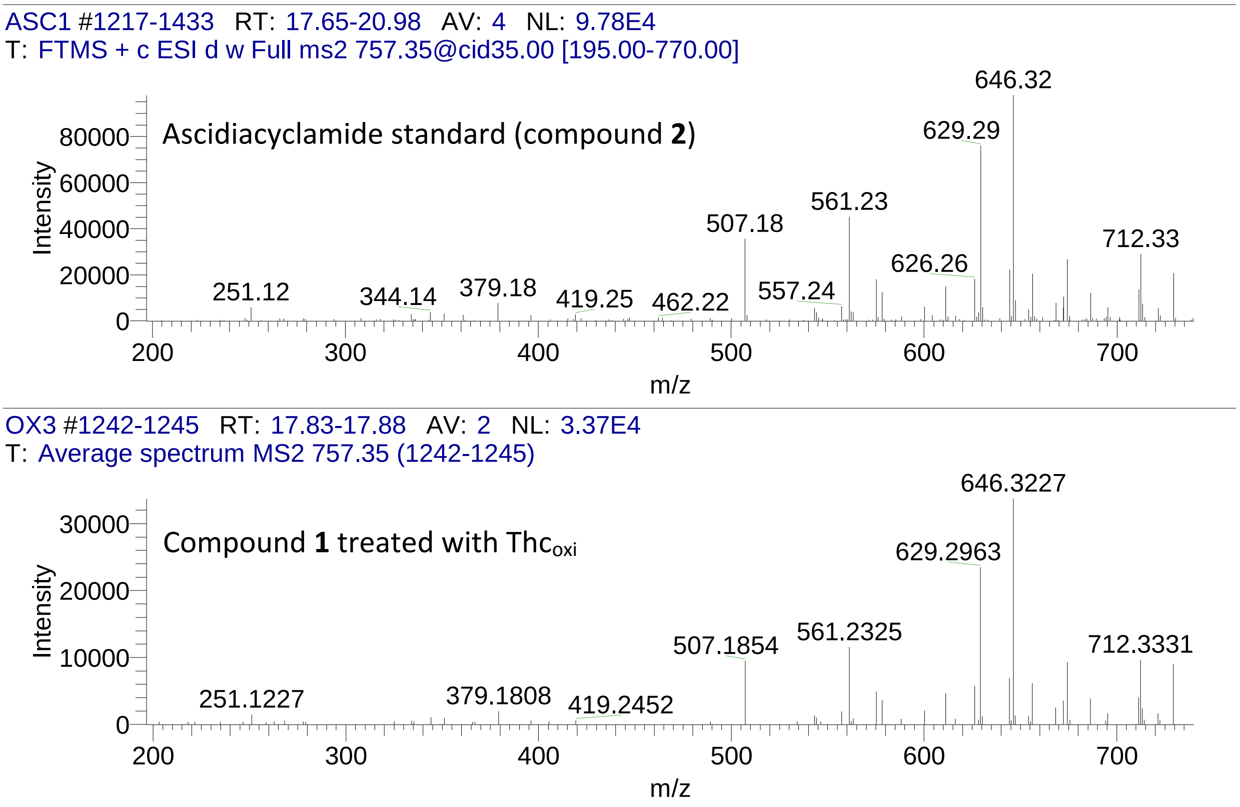


**Figure S7**. Oxidation of the cyclo [I(MeOxH)V(ThH)I(MeOxH)V(ThH)] (**1**) by $Thc_{oxi}$. a.) accurate mass measurement of compound **1** treated with $Thc_{oxi}$. b.) Comparison of MS2 fragments of ascidiacyclamide standard (**2**) and compound **1** treated with $Thc_{oxi}$.

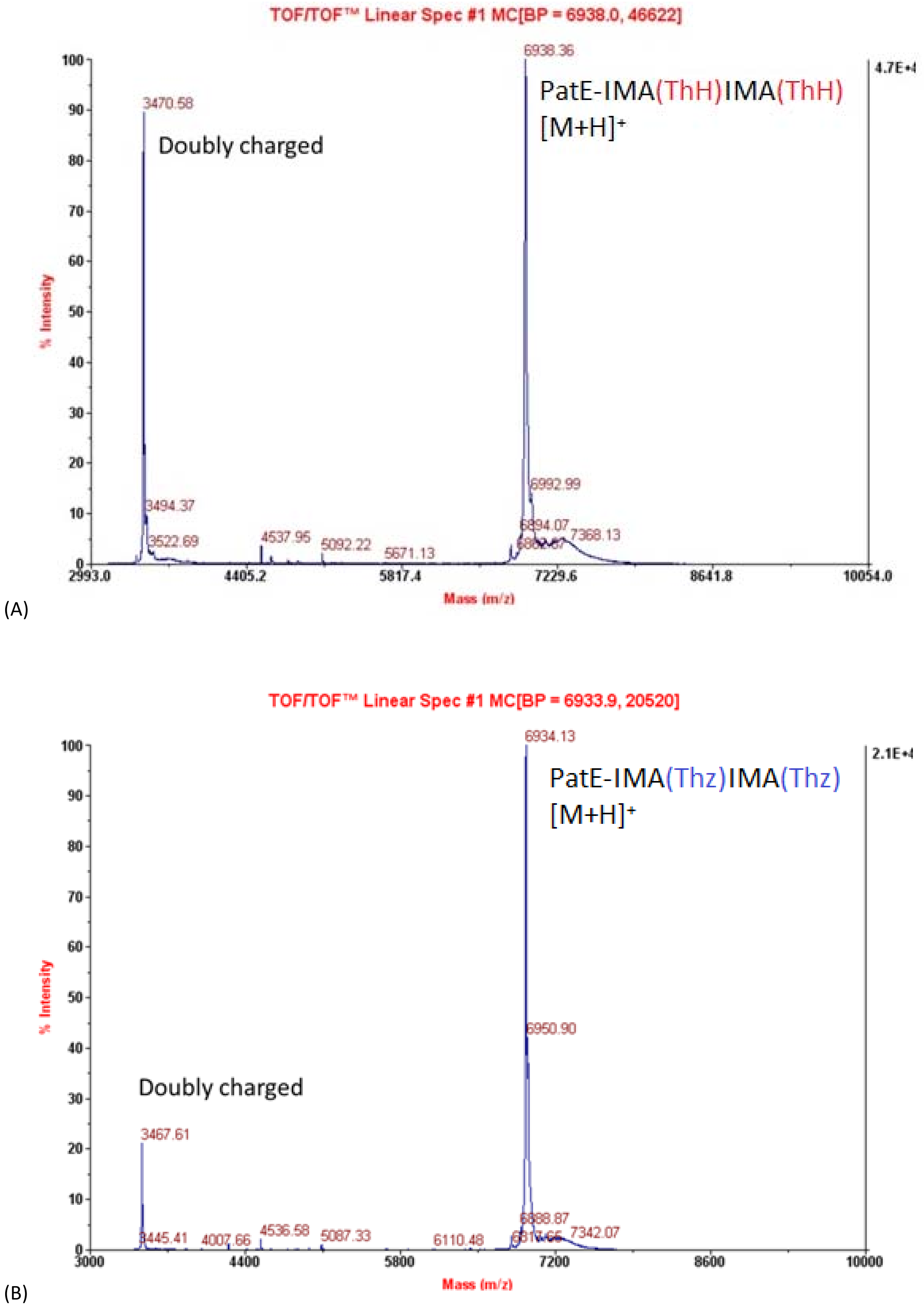


**Figure S8.** Linear oxidation of heterocycles within full length PatE by $Ap_{oxi}$. MALDI-MS of (A) heterocyclized full length $PatE_{IMACIMAC}$ prior to oxidase treatment and (B) heterocyclized and oxidized full length $PatE_{IMACIMAC}$ after oxidation reaction with $Ap_{oxi}$

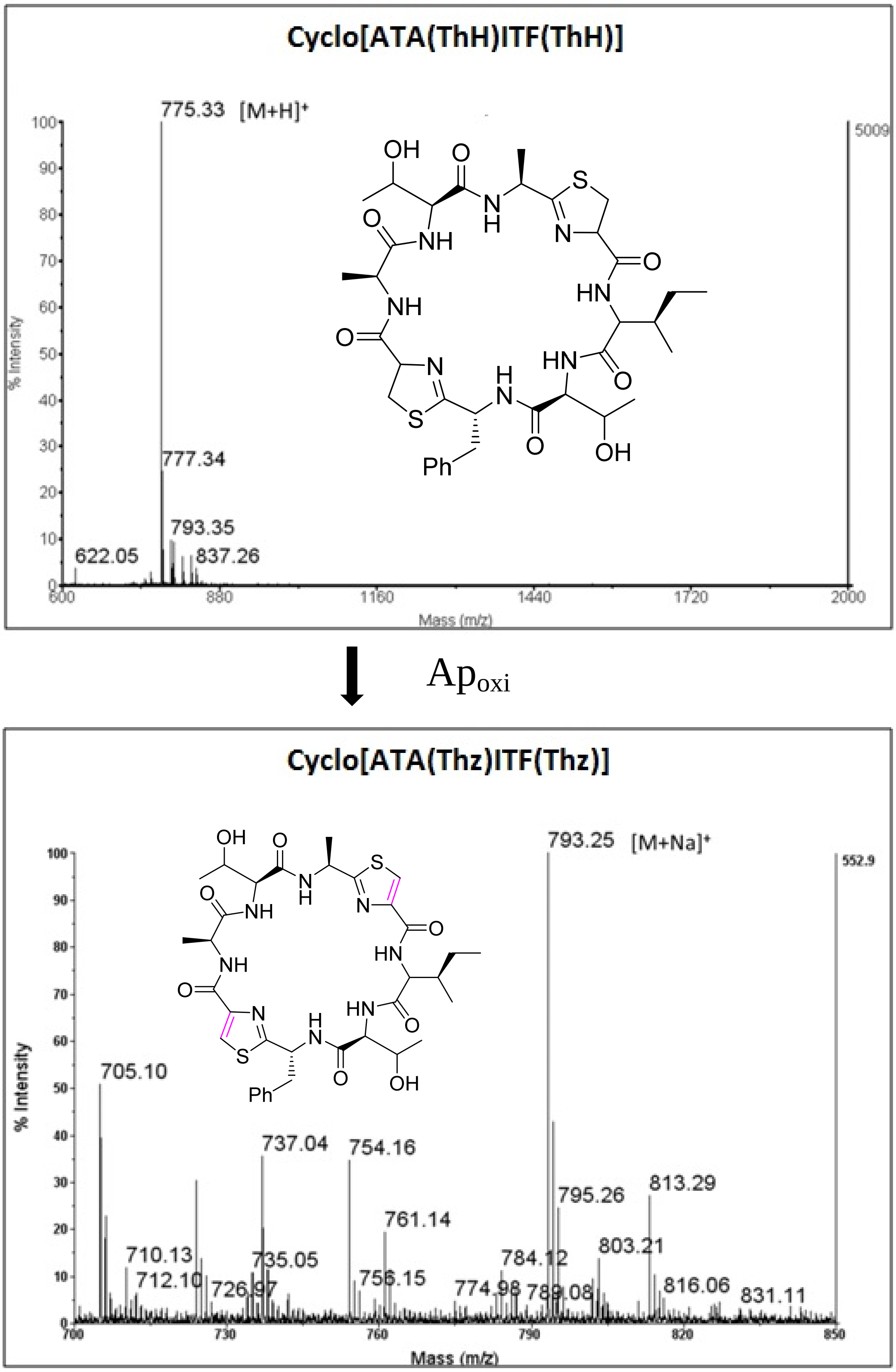


**Figure S9**. Oxidation of the cyclo [ATA(ThH)ITF(ThH)] (**6**) by $Ap_{oxi}$ as analyzed by MALDI-MS.

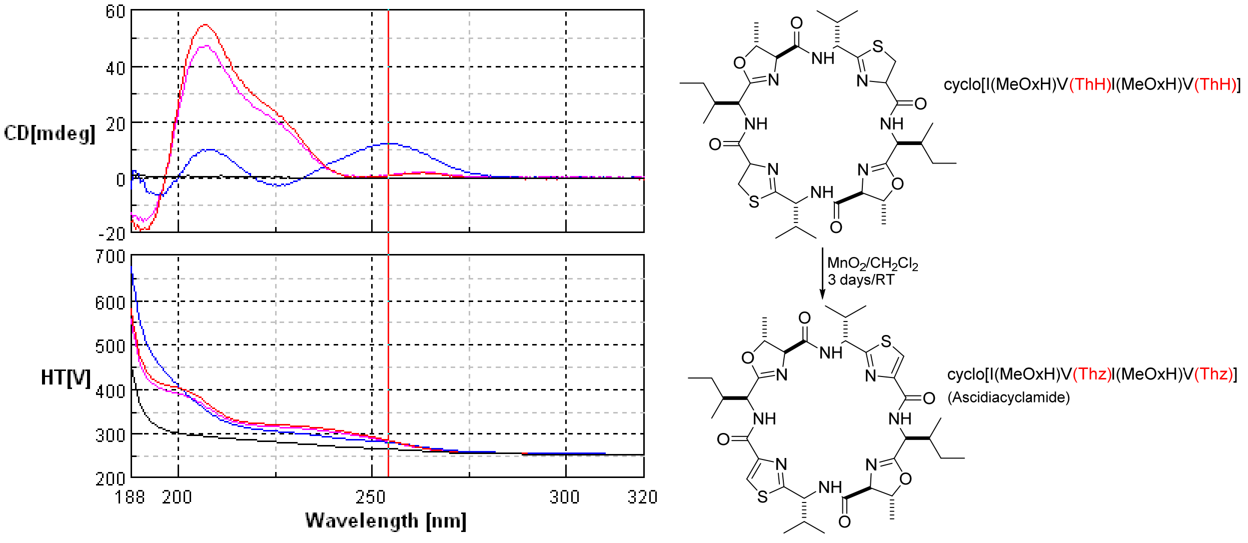


**Figure S10:** Chemical oxidation of compound **1** (natural). Far UV CD spectra of compound **1** (blue), its oxidised product (**2**) (pink), ascidiacyclamide (red) and 100% MeOH (black). Spectra were recorded at room temperature in a 0.02cm pathlength quartz cuvette using notional concentrations of ~1 mg/ml

## Spectroscopic data for cyanobactins produced *in vitro*.

Compound **1**: Precursor peptide is Leader-GLEAS-K-ITVCITVC-AYDGELE-Tag treated with PatD, trypsin and $PatG_{mac}$.

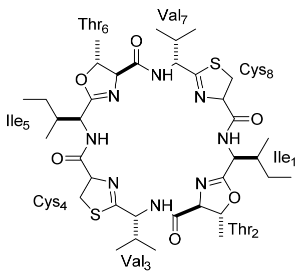


Cyclo [I(MeOxH)V(ThH)I(MeOxH)V(ThH)]

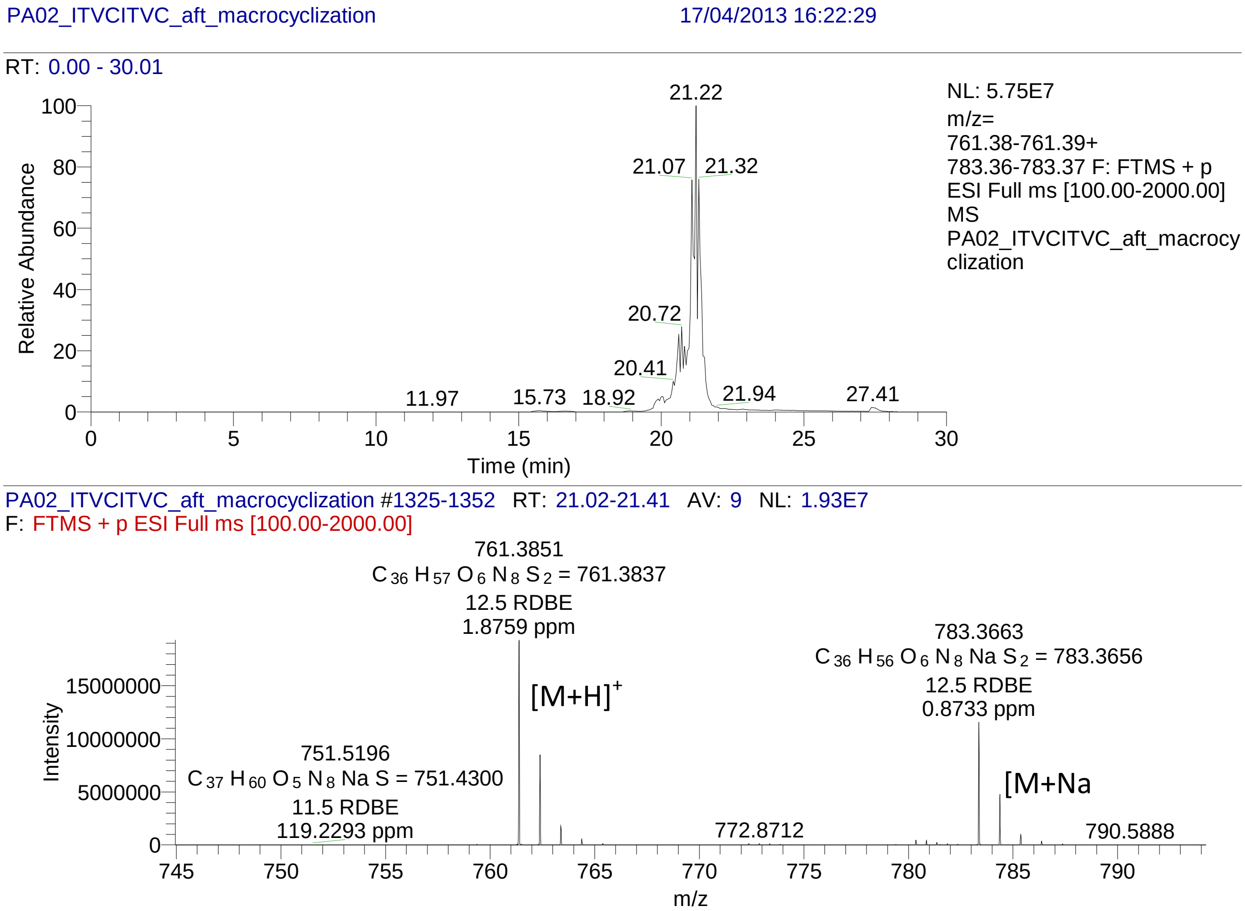


**Figure S11.** Accurate LC-MS data for compound **1**.

**Table S1.** $^1H/^{13}C$ NMR data in $CDCl_3$ at 600/150 MHz for compound **1** isolated from *Lissoclinum patella* and that obtained from *in vitro* biosynthesis.

| | | **Natural** | ***In vitro*** | **Natural** | ***In vitro*** |
|---|---|---|---|---|---|
| **Residue/Atom** | | **$\delta_C$/ppm** | **$\delta_C$/ppm** | **$\delta_H$/ppm** | **$\delta_H$/ppm** |
| **Ile1/Ile5** | | | | | |
| α | CH | 51.2 | 51.1 | 4.68 | 4.73 |
| β | CH | 38.5 | 38.6 | 1.81 | 1.85 |
| $\gamma_1$ | $CH_3$ | 15.2 | 15.1 | 0.84 | 0.89 |
| $\gamma_2$ | $CH_2$ | 24.7 | 25.2 | 1.41/1.08 | 1.46/1.11 |
| δ | $CH_3$ | 11.2 | 11.3 | 0.83 | 0.88 |
| **C=N** | C | 169.4 | 170.0 | - | - |
| **NH** | - | - | - | 7.42 | 7.39 |
| | | | | | |
| **Thr2/Thr6** | | | | | |
| α | CH | 74.3 | 74.3 | 4.21 | 4.22 |
| β | CH | 80.6 | 80.6 | 4.85 | 4.85 |
| γ | $CH_3$ | 21.8 | 21.7 | 1.46 | 1.50 |
| **C=O** | C | 170.7 | 170.8 | - | - |
| | | | | | |
| **Val3/Val7** | | | | | |
| α | CH | 55.4 | 55.1 | 4.78 | 4.81 |
| β | CH | 32.0 | 32.1 | 2.12 | 2.14 |
| $\gamma_1$ | $CH_3$ | 19.3 | 19.3 | 0.97 | 0.98 |
| $\gamma_2$ | $CH_3$ | 16.6 | 17.0 | 0.86 | 0.91 |
| **C=N** | C | 174.0 | 174.7 | - | - |
| **NH** | - | - | - | 7.24 | 7.22 |
| | | | | | |
| **Cys4/Cys8** | | | | | |
| α | CH | 77.3 | 78.6 | 5.10 | 5.13 |
| β | $CH_2$ | 35.9 | 35.6 | 3.64 | 3.68 |
| **C=O** | C | 170.9 | 170.7 | - | - |

a)

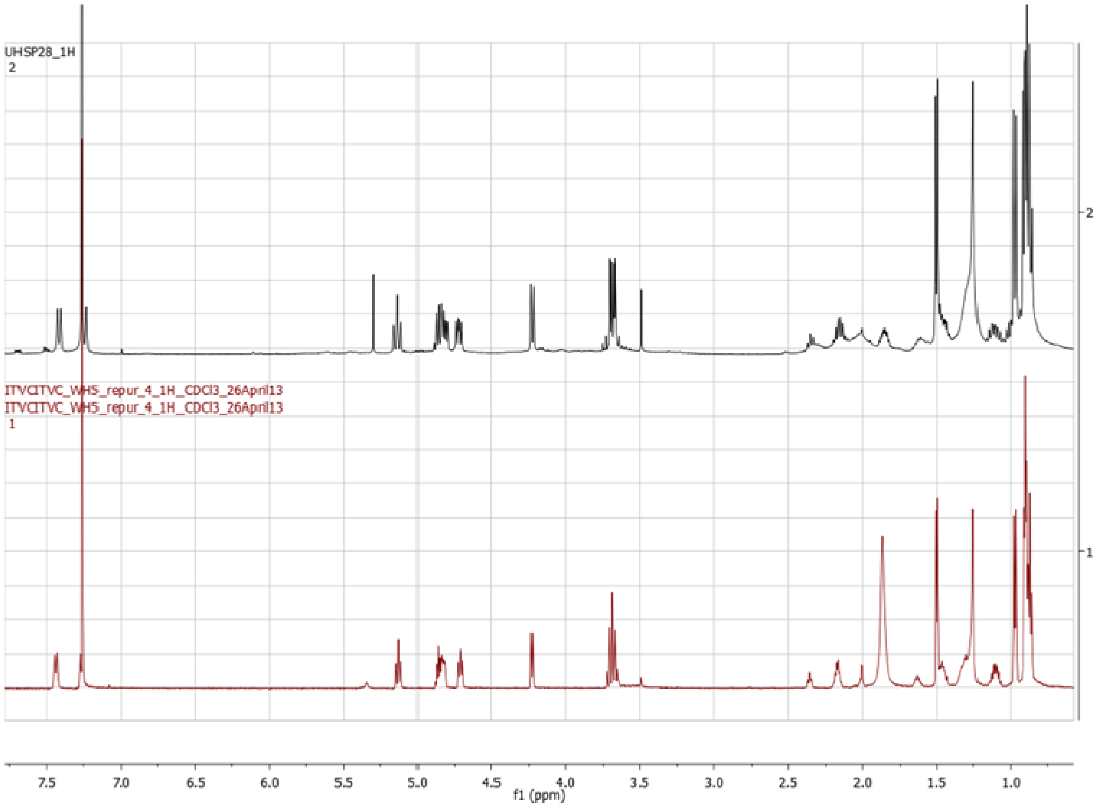


b)

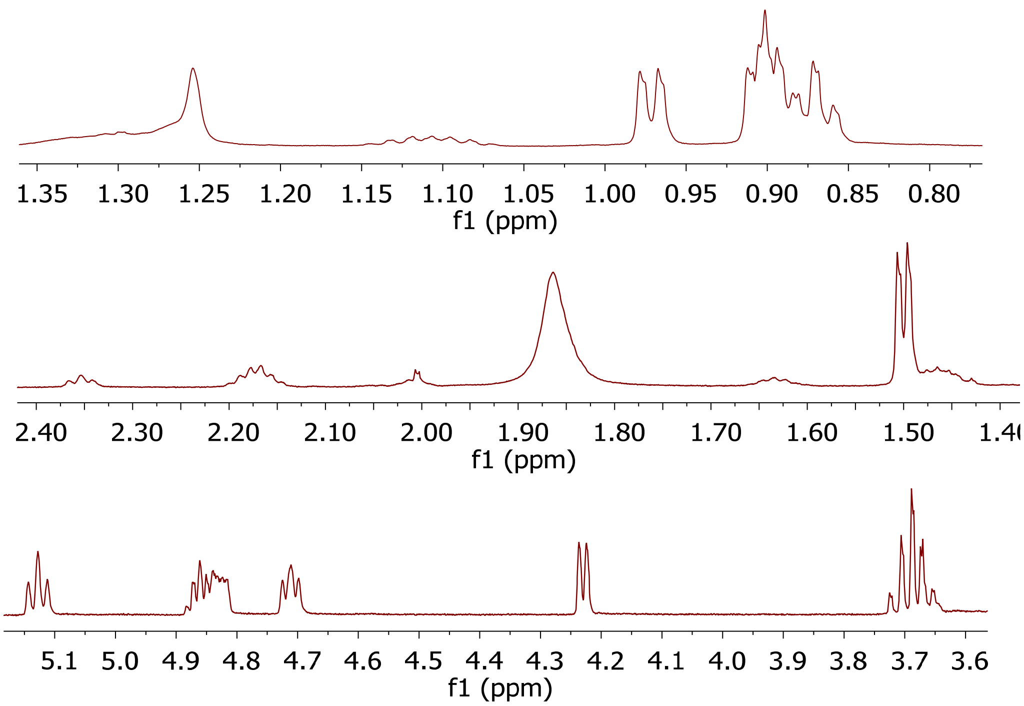


**Figure S12.** a) A comparison of the $^{1}$H NMR spectra (600 MHz, $CDCl_3$) of natural compound **1** (top, black) and compound **1** using the *in vitro* biosynthetic method (bottom, red). Peaks at $\delta_H$ 1.9, 5.3 and 3.5 ppm in the NMR of the synthetic material correspond to traces of water, dichloromethane and methanol respectively in the sample. b) A zoomed in $^{1}$H NMR spectrum of the synthesised compound **1**.

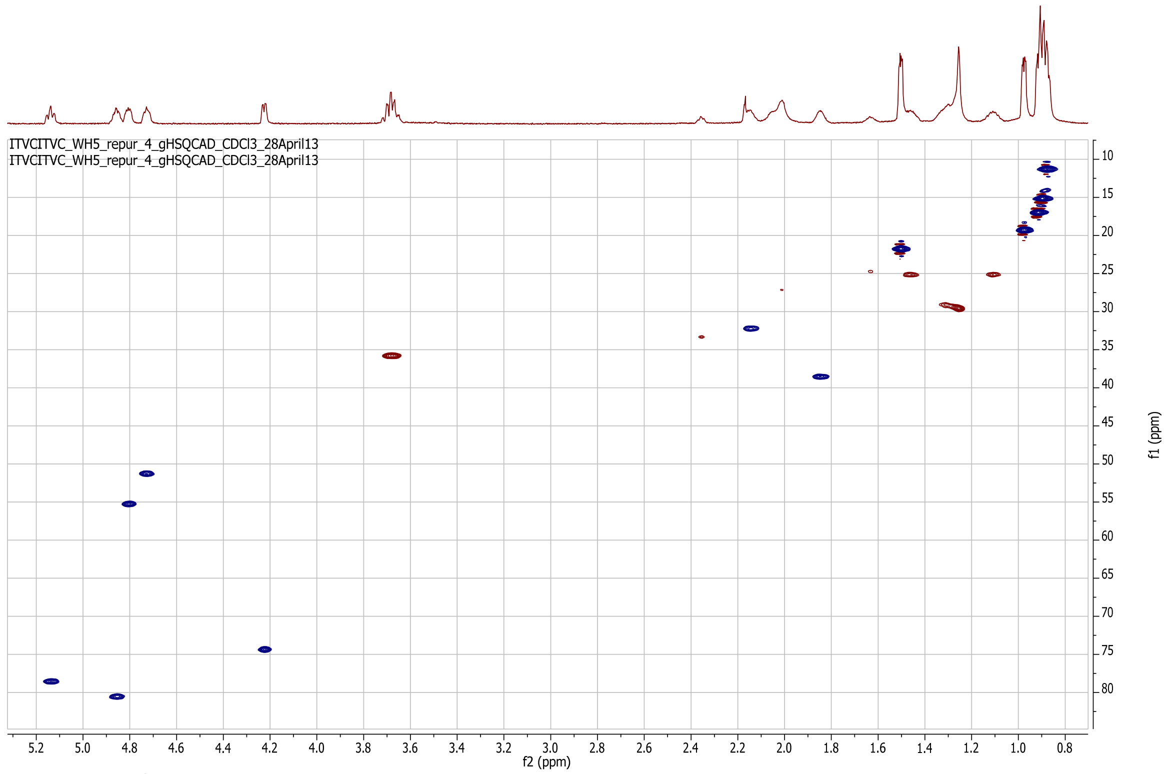


**Figure S13.** $^{1}H$-$^{13}C$ HSQC NMR spectrum of compound **1** at 600/150 MHz in $CDCl_3$.

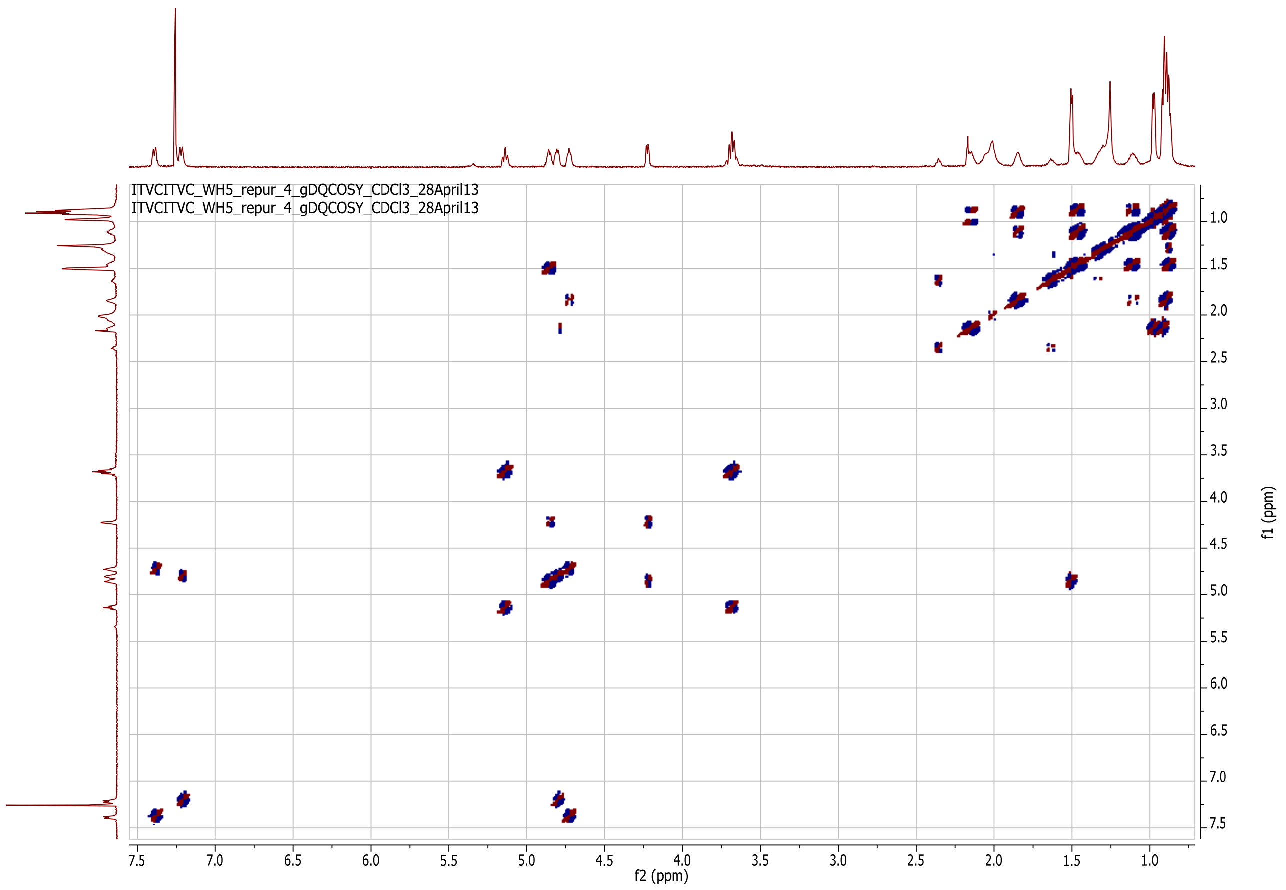


**Figure S14.** $^{1}H$-$^{1}H$ DQF-COSY NMR spectrum of compound **1** at 600/150 MHz in $CDCl_3$.

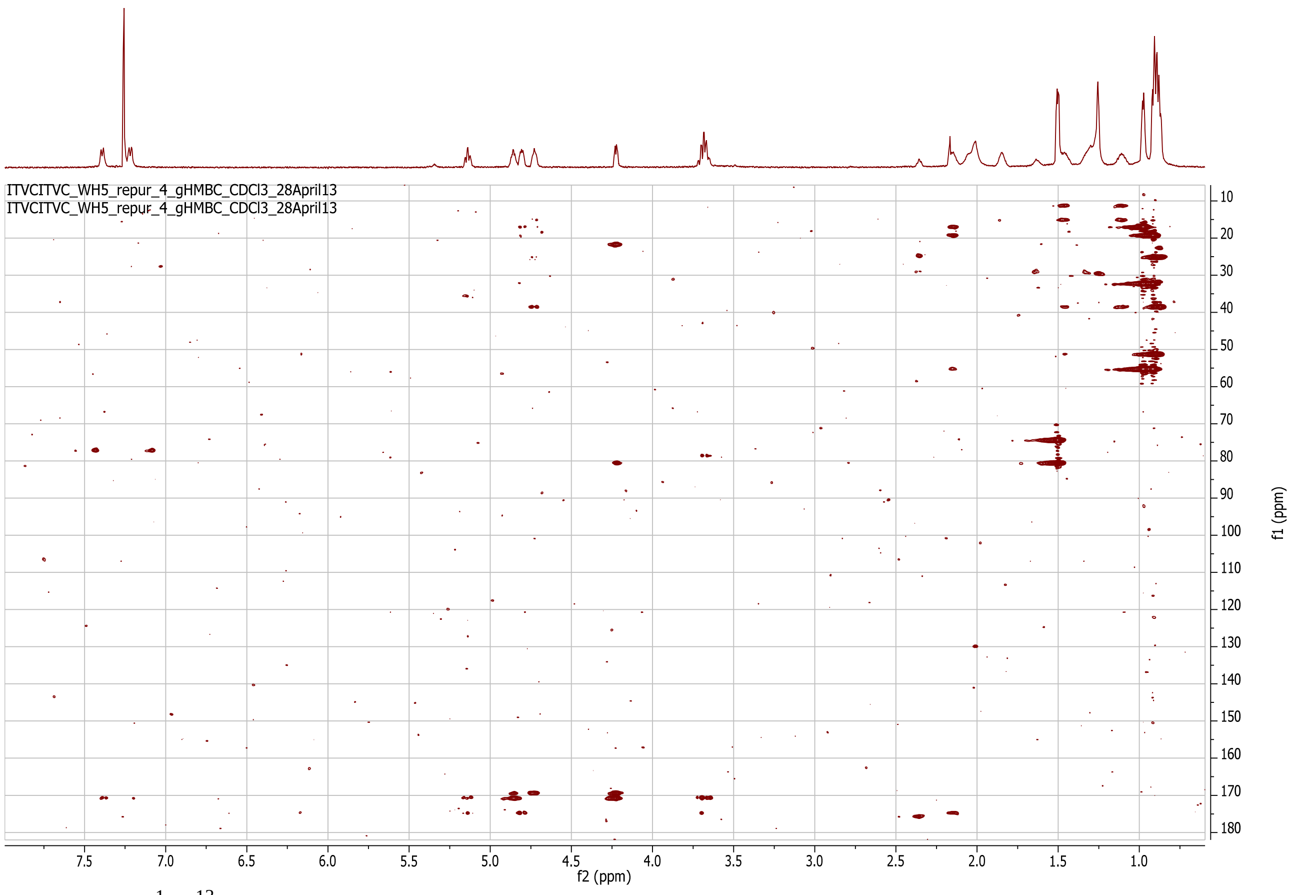


**Figure S15.** $^{1}H$-$^{13}C$ HMBC NMR spectrum of compound **1** at 600/150 MHz in $CDCl_3$.

<u>Compound **3**:</u> Precursor peptide is Leader-GLEAS-K-<u>ITACITFC</u>-AYDGELE-Tag treated with TruD, trypsin and $PatG_{mac}$.

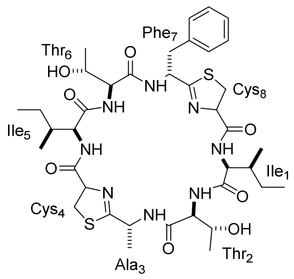


Cyclo[ITA(ThH)ITF(ThH)]

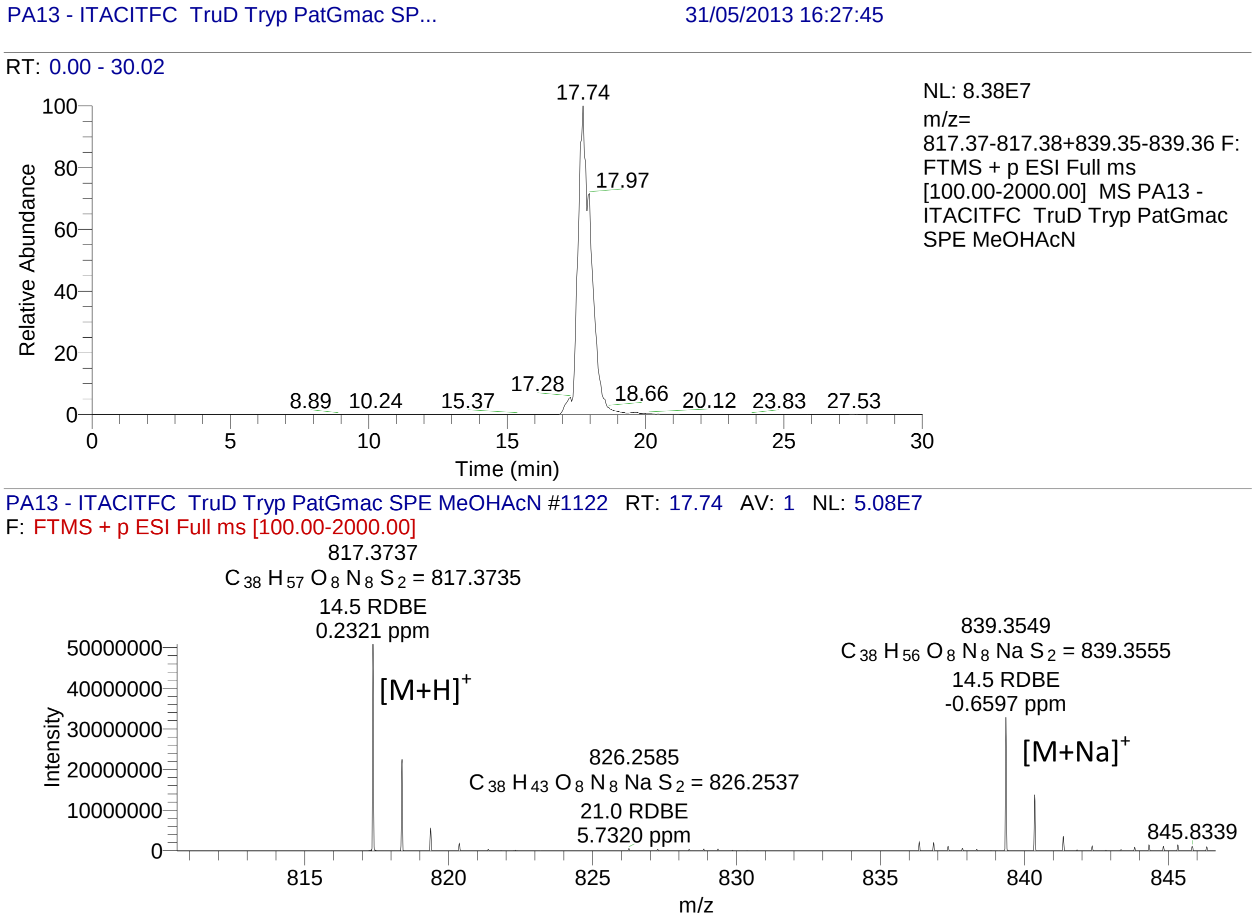


**Figure S16.** Accurate LC-MS data for compound **3**.

**Table S2.** $^1H/^{13}C$ NMR data in $CDCl_3$ at 600/150 MHz for compound **3** obtained from *in vitro* biosynthesis.

| Residue/Atom | | ***In vitro*** $\delta_C$/ppm | ***In vitro*** $\delta_H$/ppm |
|---|---|---|---|
| **Ile1[a]** | | | |
| α | CH | 56.7 | 4.52 |
| β | CH | 38.4 | 1.76 |
| $\gamma_1$ | $CH_3$ | 15.3 | 0.88 |
| $\gamma_2$ | $CH_2$ | 25.0 | 1.48/1.12 |
| δ | $CH_3$ | 11.0 | 0.89 |
| **C=O** | C | n.o. | - |
| **NH** | - | - | 7.56 |
| **Thr2[b]** | | | |
| α | CH | 56.6 | 4.32 |
| β | CH | 65.1 | 4.44 |
| γ | $CH_3$ | 18.4 | 1.14 |
| **C=O** | C | 172.6 | - |
| **NH** | - | - | 7.07 |
| **Ala3** | | | |
| α | CH | 48.4 | 4.69 |
| β | CH | 20.7 | 1.37 |
| **C=N** | C | 174.9 | - |
| **NH** | - | 8.48 | - |
| **Cys4** | | | |
| α | CH | 77.6 | 5.05 |
| β | $CH_2$ | 36.6 | 3.70 |
| **C=O** | C | 170.3 | - |
| **Ile5[a]** | | | |
| α | CH | 56.8 | 4.53 |
| β | CH | 38.4 | 1.76 |
| $\gamma_1$ | $CH_3$ | 14.1 | 0.85 |
| $\gamma_2$ | $CH_2$ | 25.0 | 1.45/1.10 |
| δ | $CH_3$ | 11.0 | 0.83 |
| **C=O** | C | n.o. | - |
| **NH** | - | - | 7.42 |
| **Thr6[b]** | | | |
| α | CH | 56.2 | 4.30 |
| β | CH | 65.1 | 4.41 |
| γ | $CH_3$ | 17.27 | 1.10 |
| **C=O** | C | 170.6 | - |
| **NH** | - | - | 7.07 |
| **Phe7** | | | |
| α | CH | 54.2 | 4.86 |
| β | $CH_2$ | 40.9 | 3.18/2.72 |
| γ | C | 136.2 | - |
| δ | $(CH)_2$ | 129.2 | 7.18 |
| ε | $(CH)_2$ | 128.3 | 7.24 |
| ζ | CH | 127.1 | 7.21 |
| **C=N** | C | 173.3 | - |
| **NH** | - | - | 8.46 |
| **Cys8** | | | |
| α | CH | 77.12 | 4.94 |
| β | $CH_2$ | 36.53 | 3.57/3.44 |
| **C=O** | C | 170.2 | - |

[a/b] – Residues may be exchanged

n.o. – not observed

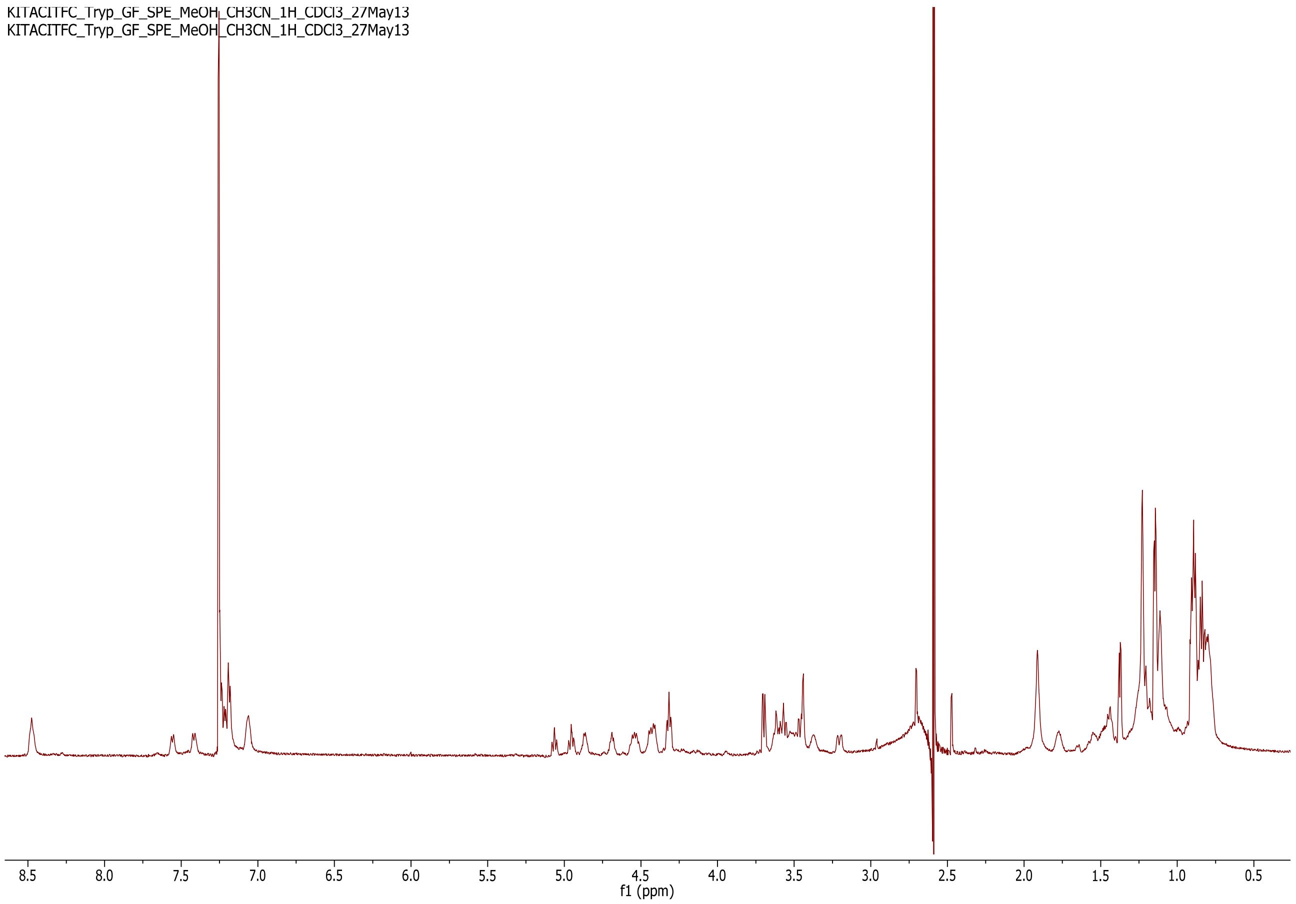


**Figure S17.** $^{1}$H NMR spectra (600 MHz, $CDCl_3$) of compound **3** generated using the *in vitro* biosynthetic method.

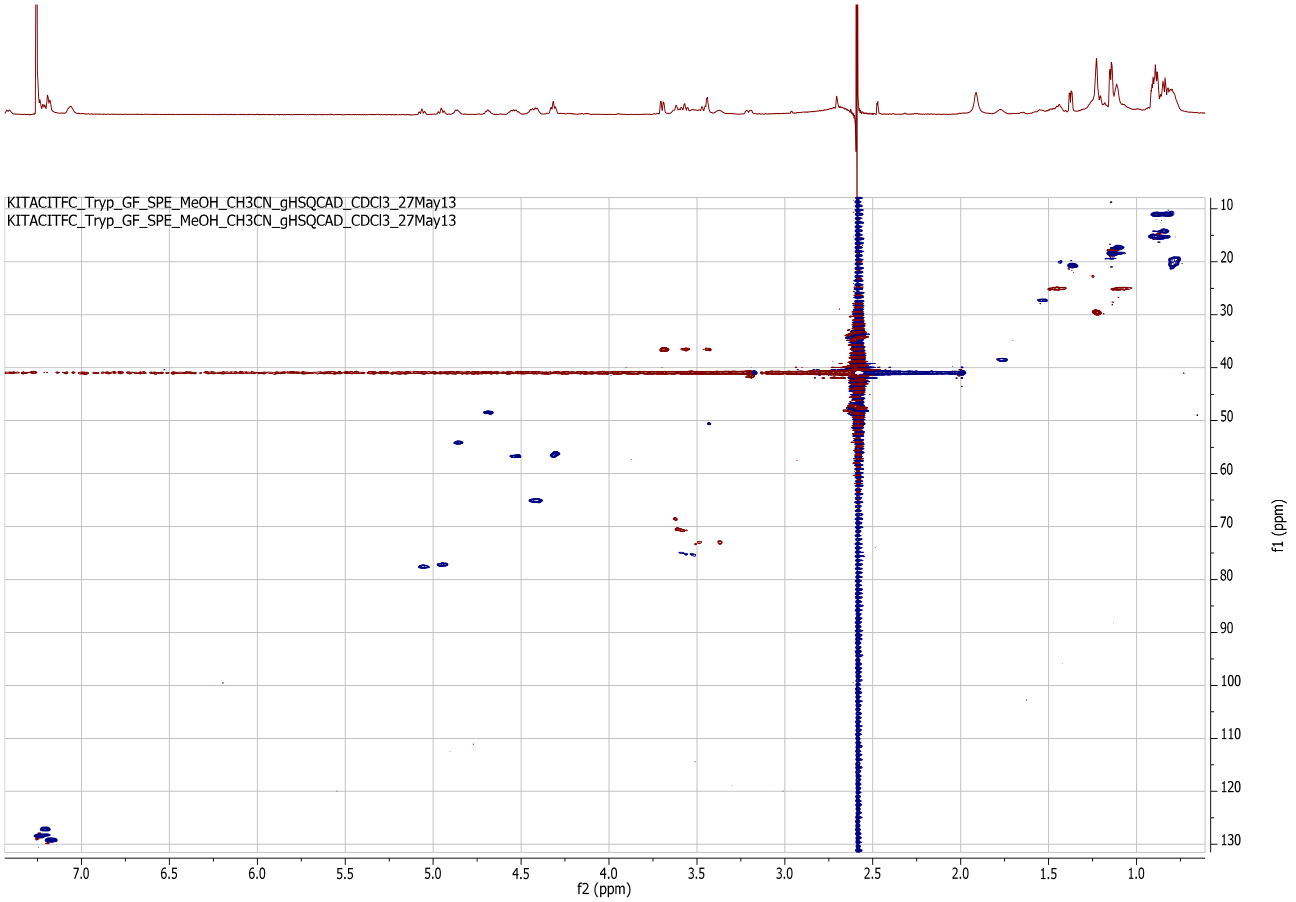


**Figure S18.** $^{1}H$-$^{13}C$ HSQC NMR spectrum of compound **3** at 600/150 MHz in $CDCl_3$.

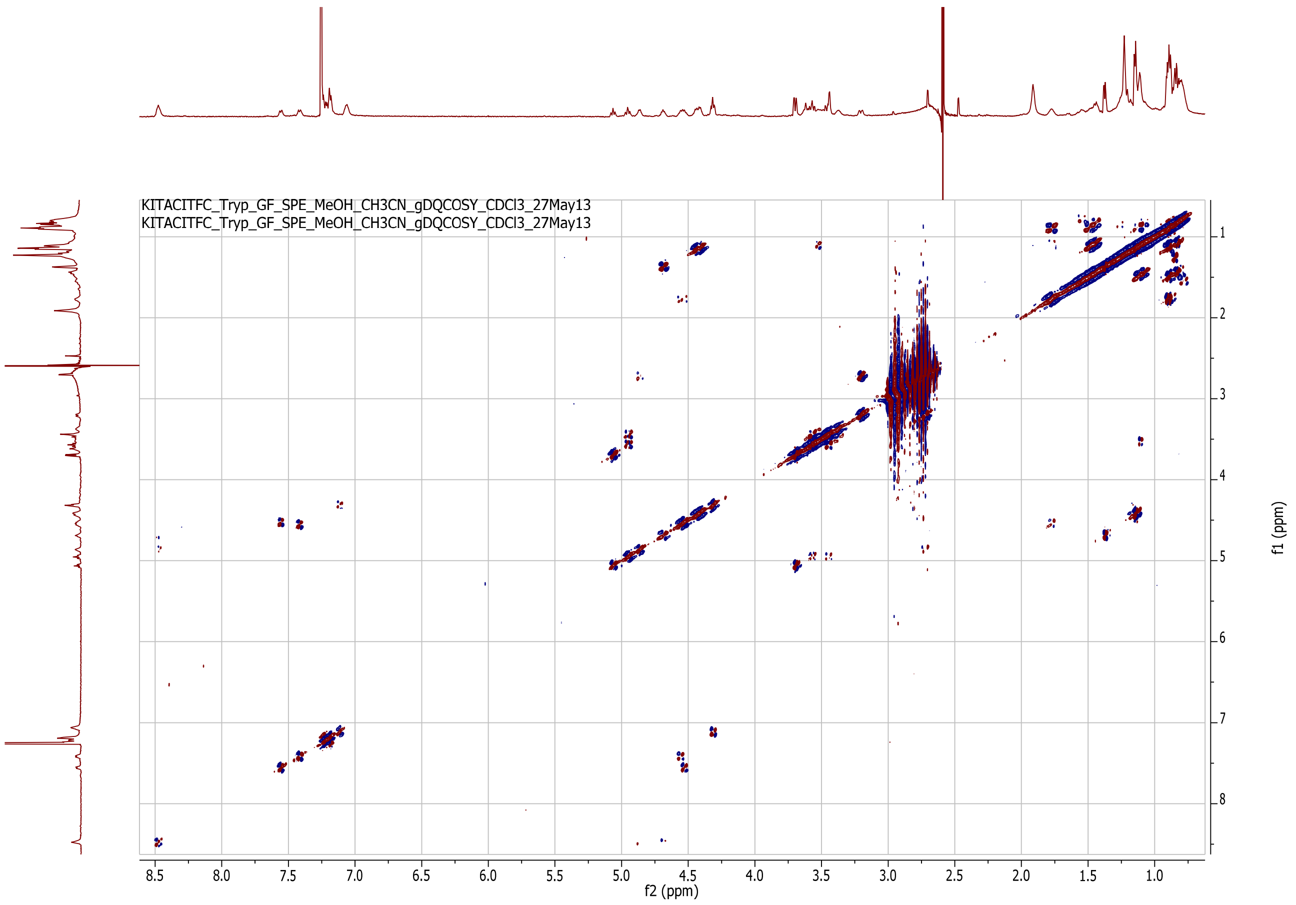


**Figure S19.** $^{1}$H-$^{1}$H DQF-COSY NMR spectrum of compound **3** at 600/150 MHz in $CDCl_3$.

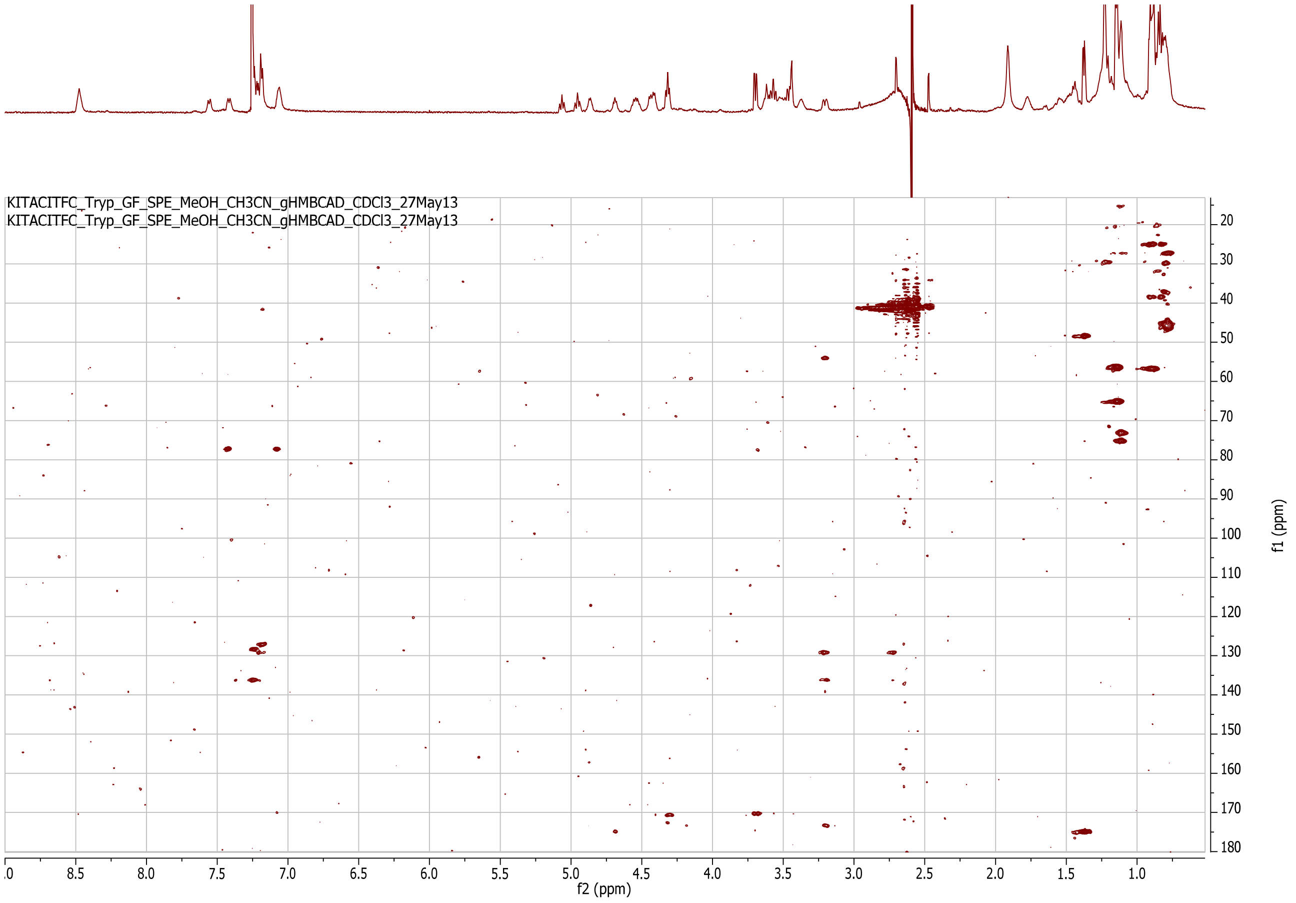


**Figure S20.** ${}^{1}H$-${}^{13}C$ HMBC NMR spectrum of compound **3** at 600/150 MHz in $CDCl_3$.

Compound 4: Precursor peptide is Leader-GLEAS-K-MTVCMTVC-AYDGELE-Tag treated with TruD, trypsin and $PatG_{mac}$.

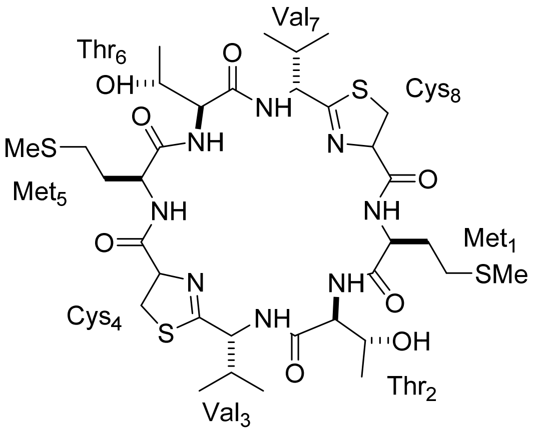


Cyclo[MTV(ThH)MTV(ThH)]

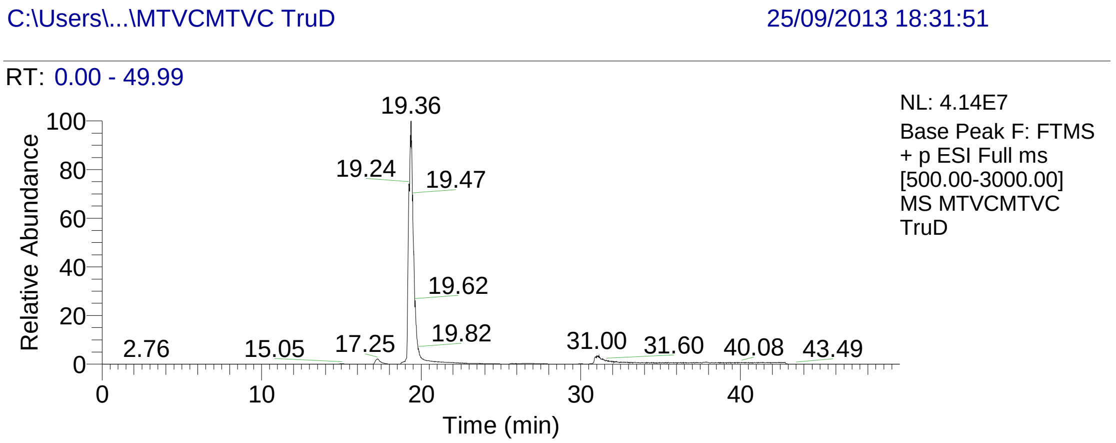


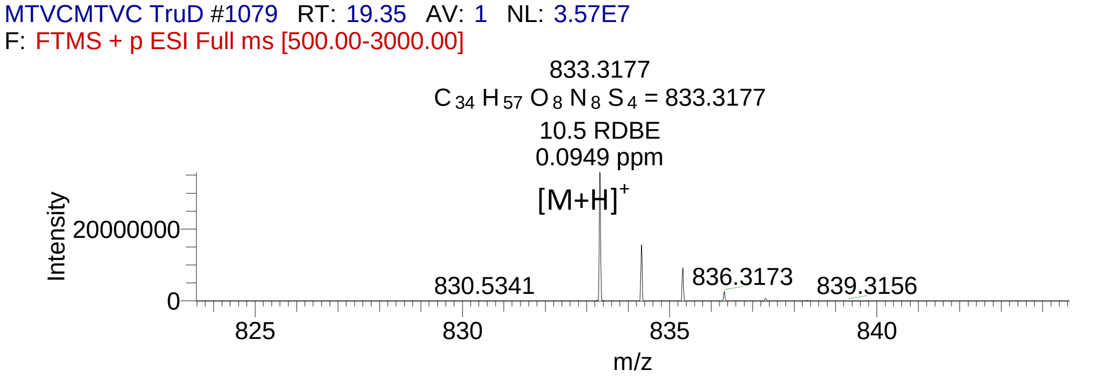


**Figure S21.** Accurate LC-MS data for compound **4**.

**Table S3.** $^{1}H/^{13}C$ NMR data in $CDCl_3$ at 600/150 MHz for compound **4** obtained from *in vitro* biosynthesis

| | | ***In vitro*** | ***In vitro*** |
|---|---|---|---|
| **Residue/Atom** | | **$\delta_C$/ppm** | **$\delta_H$/ppm** |
| **Met1/5** | | | |
| α | CH | 51.05 | 4.82 |
| β | CH | 33.85 | 1.90 |
| $\gamma_1$ | $CH_3$ | 30.04 | 2.61 |
| δ | $CH_3$ | 15.04 | 2.09 |
| **C=O** | | 172.1 | |
| **NH** | - | 7.64 | |
| | | | |
| **Thr2/Thr6** | | | |
| α | CH | 56.14 | 4.39 |
| β | CH | 65.12 | 4.45 |
| γ | $CH_3$ | 18.18 | 1.18 |
| **C=O** | C | 171.1 | |
| NH | - | 7.09 | |
| **Val3/Val7** | | | |
| α | CH | 55.80 | 4.88 |
| β | CH | 32.24 | 2.18 |
| $\gamma_1$ | $CH_3$ | 18.81 | 0.91 |
| $\gamma_2$ | $CH_3$ | 16.31 | 0.83 |
| **C=N** | C | | |
| **NH** | - | 7.89 | |
| **C=O** | C | 173.87 | |
| | | | |
| **Cys4/Cys8** | | | |
| α | CH | 77.28 | 5.1 |
| β | $CH_2$ | 36.22 | 3.48 |
| **C=O** | C | 170.42 | |

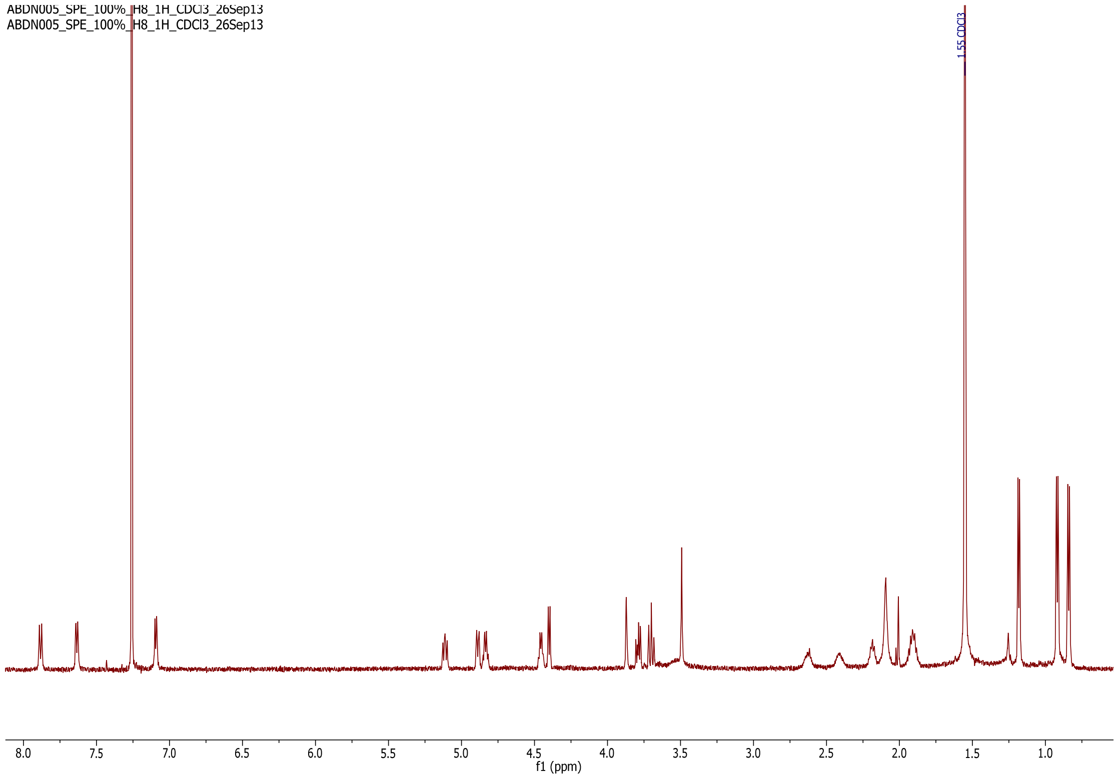


**Figure S22.** $^{1}$H NMR spectra (600 MHz, $CDCl_3$) of compound **4** generated using the *in vitro* biosynthetic method.

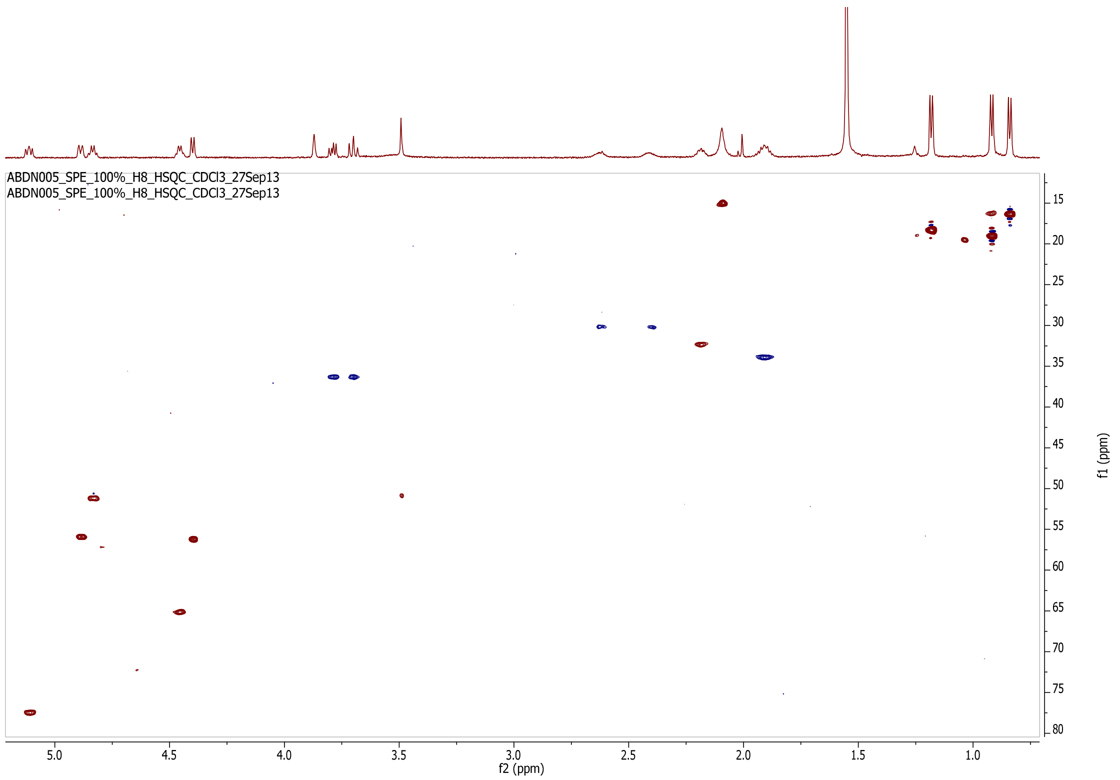


**Figure S23.** $^{1}H$-$^{13}C$ HSQC NMR spectrum of compound **4** at 600/150 MHz in $CDCl_3$.

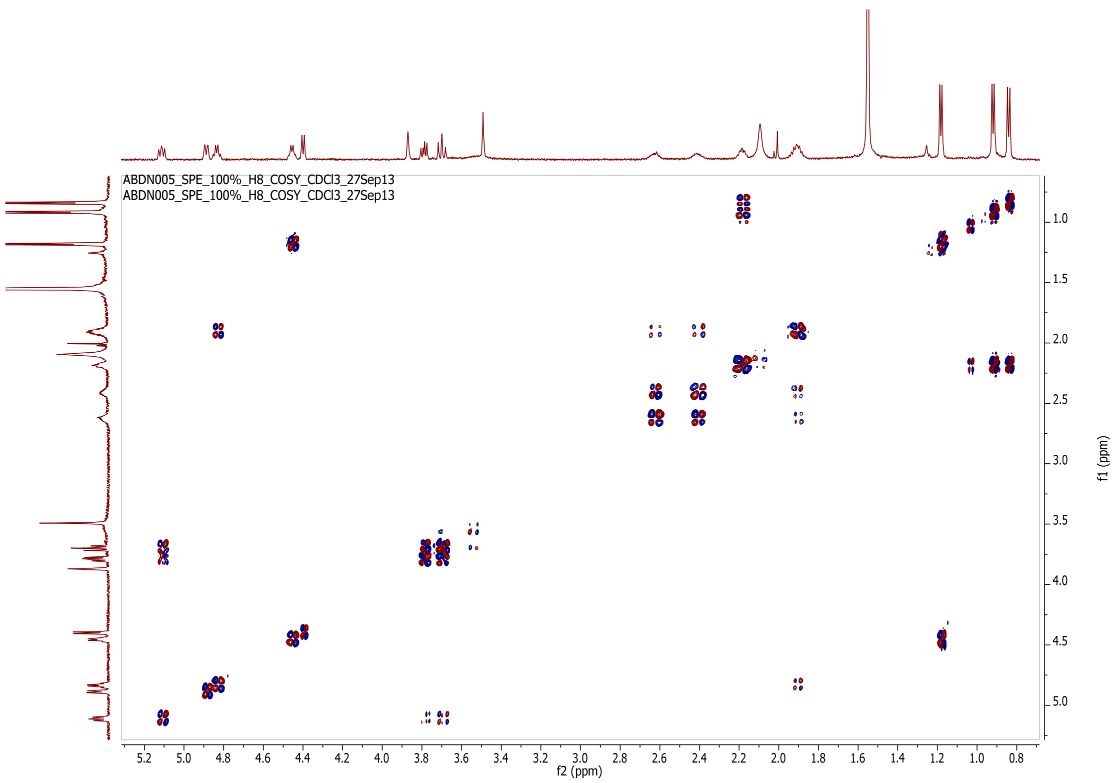


**Figure S24.** $^{1}H$-$^{1}H$ DQF-COSY NMR spectrum of compound **4** at 600/150 MHz in $CDCl_3$.

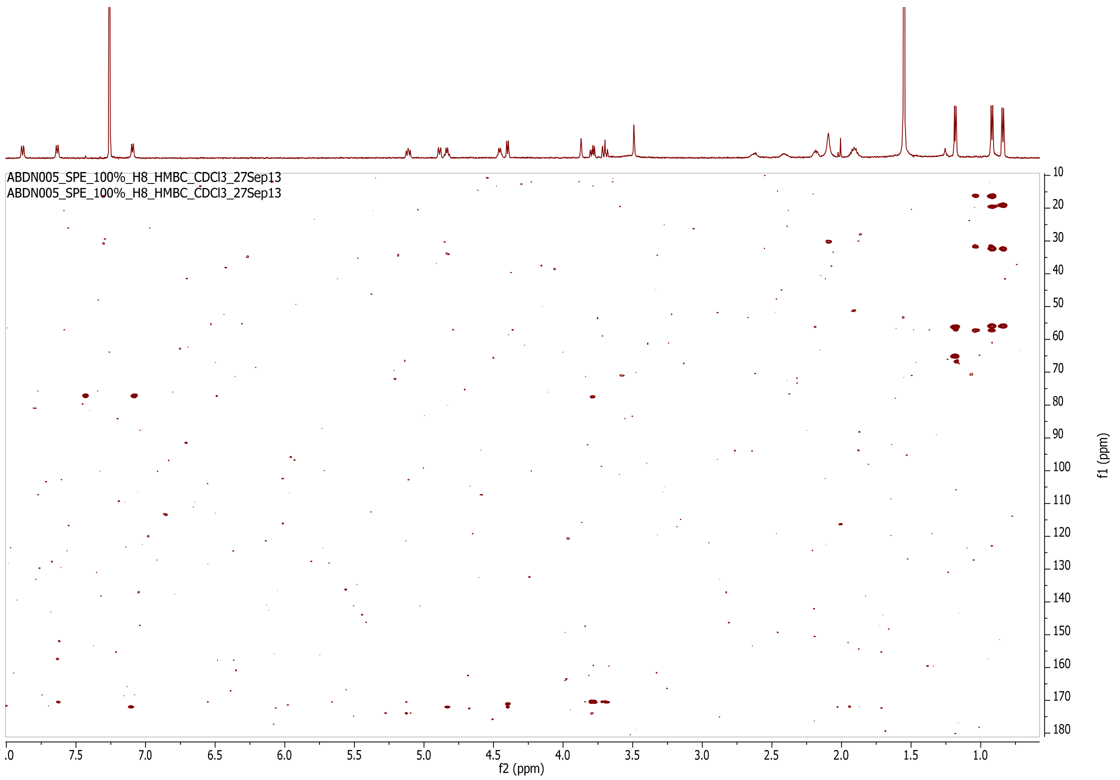


**Figure S25.** $^{1}H$-$^{13}C$ HMBC NMR spectrum of compound **4** at 600/150 MHz in $CDCl_3$.

Compound 5: Precursor peptide is Leader-GLEAS-K-ATACITFC-AYDGELE-Tag treated with TruD, trypsin and PatG$_{mac}$.

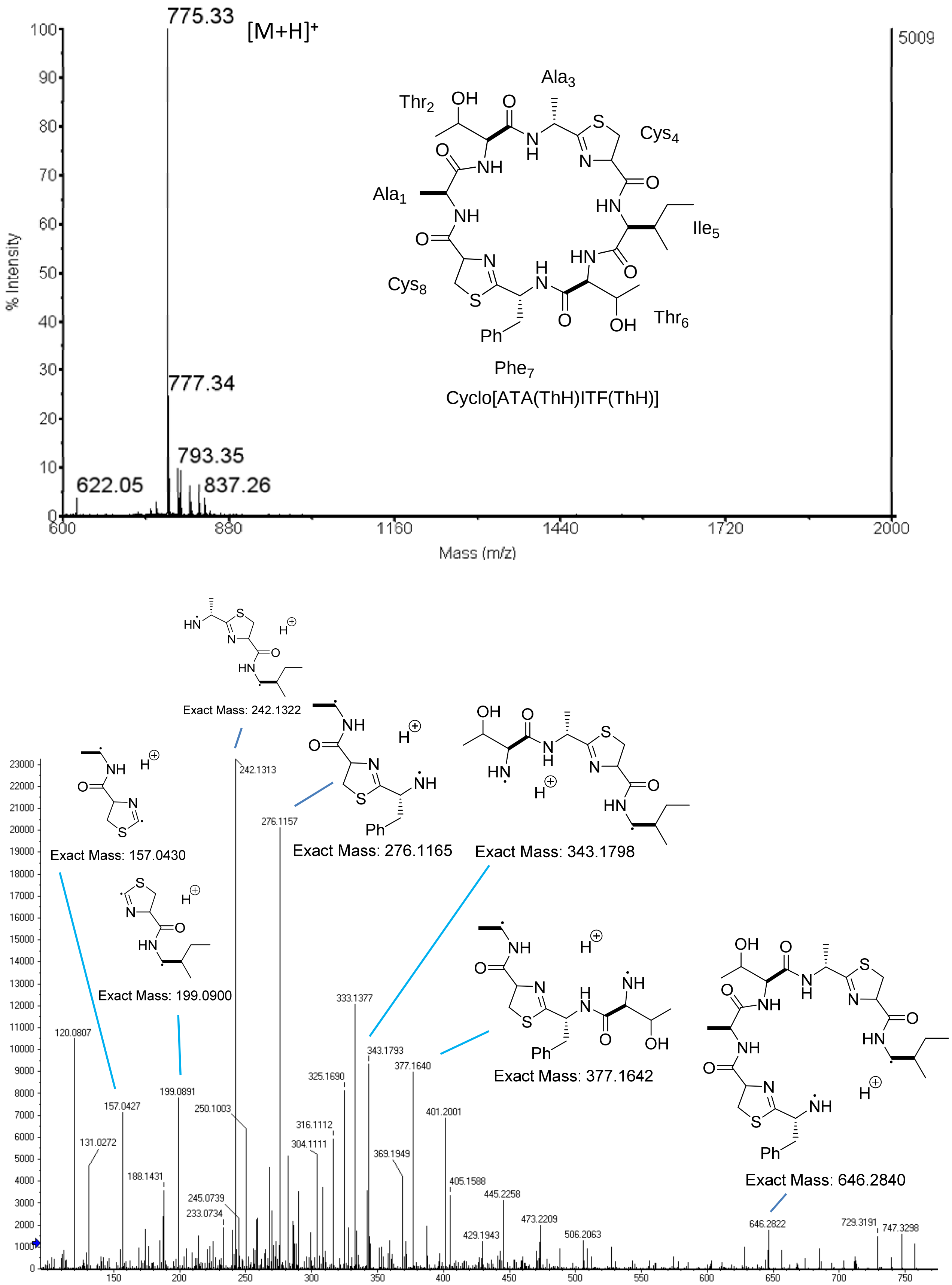


**Figure S26.** MALDI-MS and MS-MS data for compound **5**.

Compound 7: Precursor peptide is Leader-GLEAS-K-IMACIMAC-AYDGELE-Tag treated with TruD, trypsin and $PatG_{mac}$.

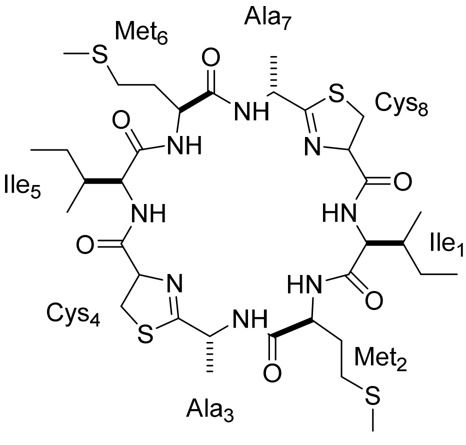


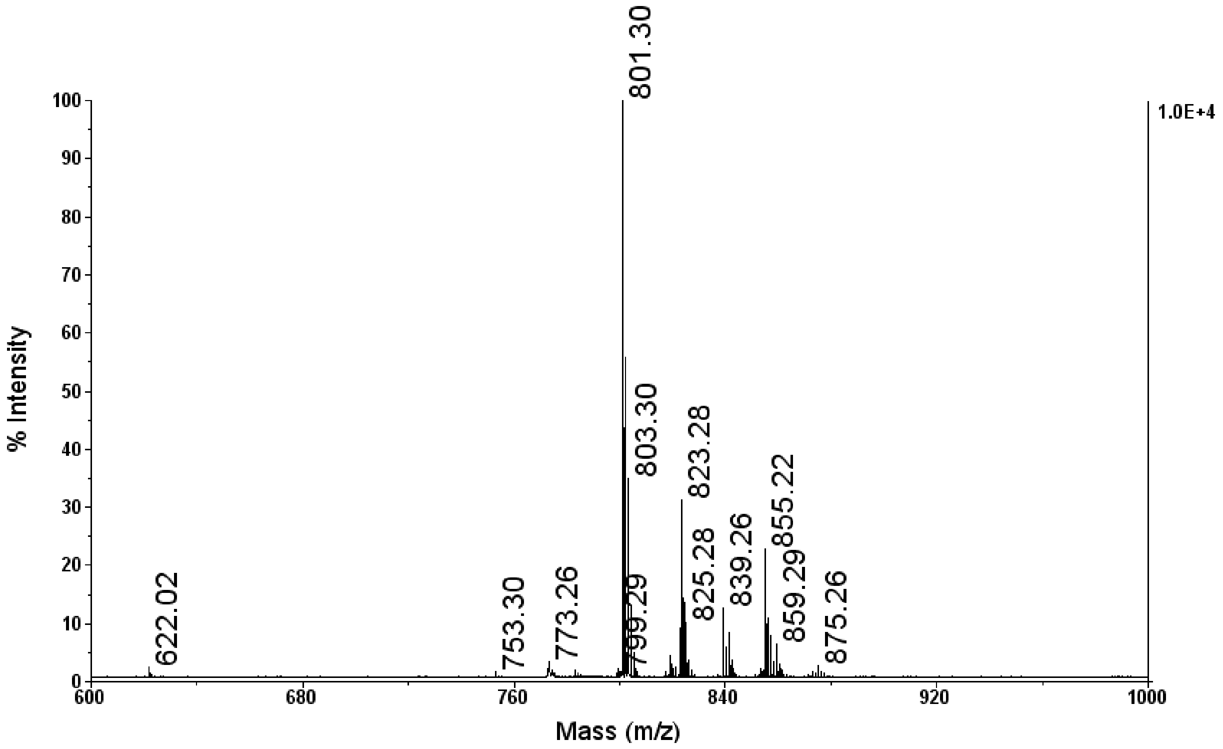


**Figure S27.** MALDI-MS data for compound **7**.

**Table S4.** $^{1}$H NMR data in $CD_3OD$ at 500 MHz for compound **7** obtained from *in vitro* biosynthesis.

| **Residue/Atom** | **$\delta_H$/ppm** |
|---|---|
| **Ile1/Ile5** | |
| α | 4.27 |
| β | 1.71 |
| $\gamma_1$ | 0.87 |
| $\gamma_2$ | 1.38/1.06 |
| δ | 0.80 |
| | |
| **Met2/Met6** | |
| α | 4.51 |
| β | 2.01/1.87 |
| γ | 2.48 |
| Me | 2.03 |
| | |
| **Ala3/Ala7** | |
| α | 4.56 |
| β | 1.40 |
| | |
| **Cys4/Cys8** | |
| α | 5.08 |
| β | 3.68/3.44 |

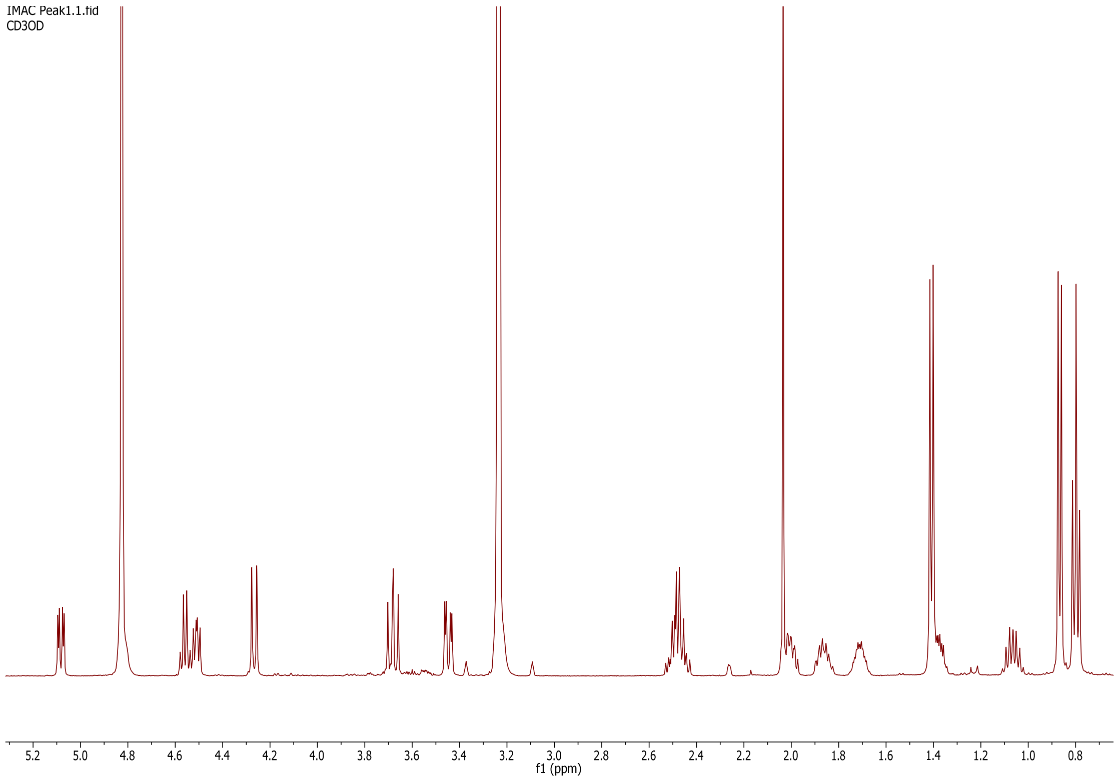


**Figure S28.** $^{1}H$ NMR spectrum (500 MHz, $CD_3OD$) of compound **7** generated using the *in vitro* biosynthetic method.

Compound 8: Precursor peptide is Leader-GLEAS-K-ITACITAC-AYDGELE-Tag treated with TruD, trypsin and $PatG_{mac}$.

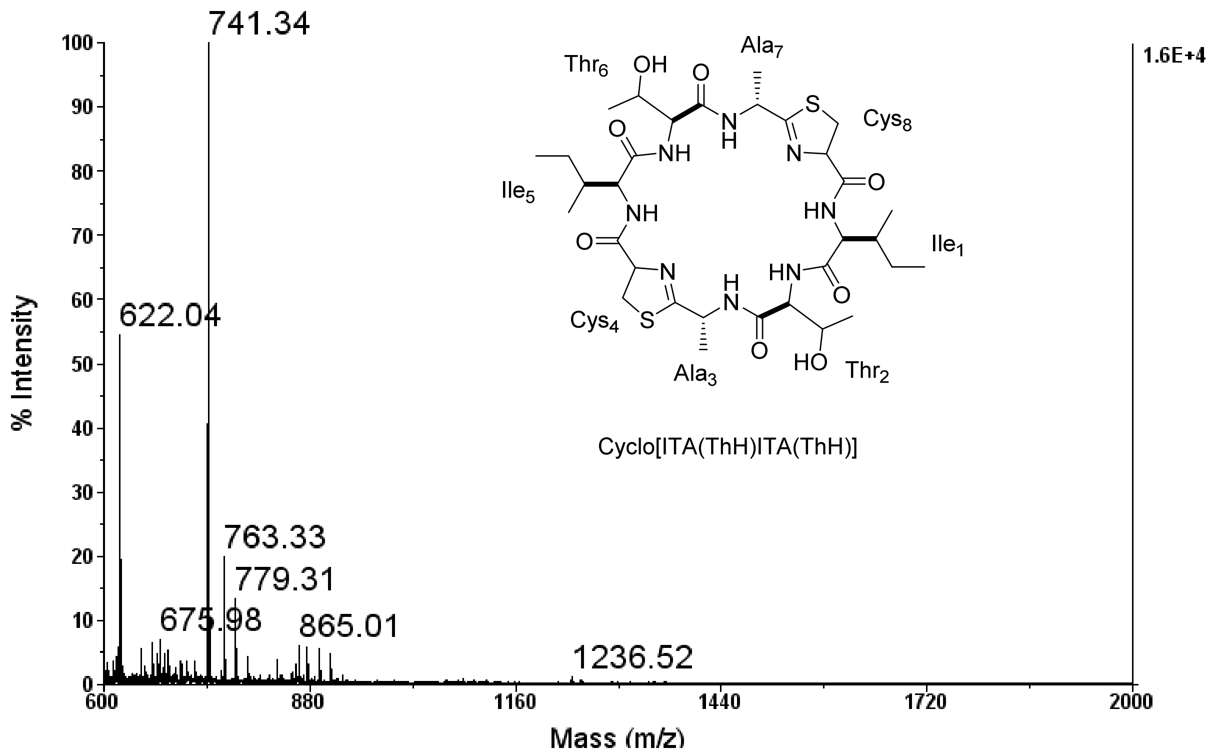


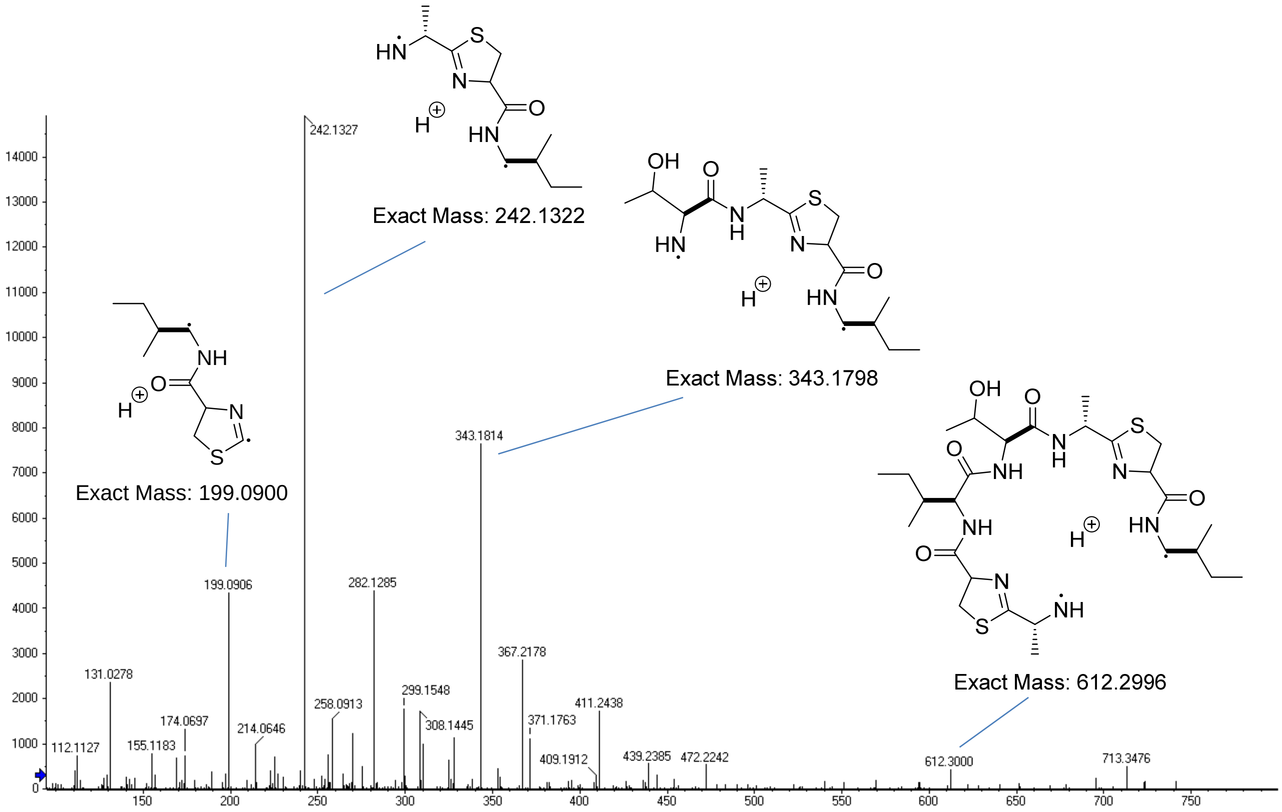


**Figure S29.** MALDI-MS and MS-MS data for compound **8**.

Compound **9**: Precursor peptide is Leader-GLEAS-K-ITACISFC-AYDGELE-Tag treated with TruD, trypsin and $PatG_{mac}$.

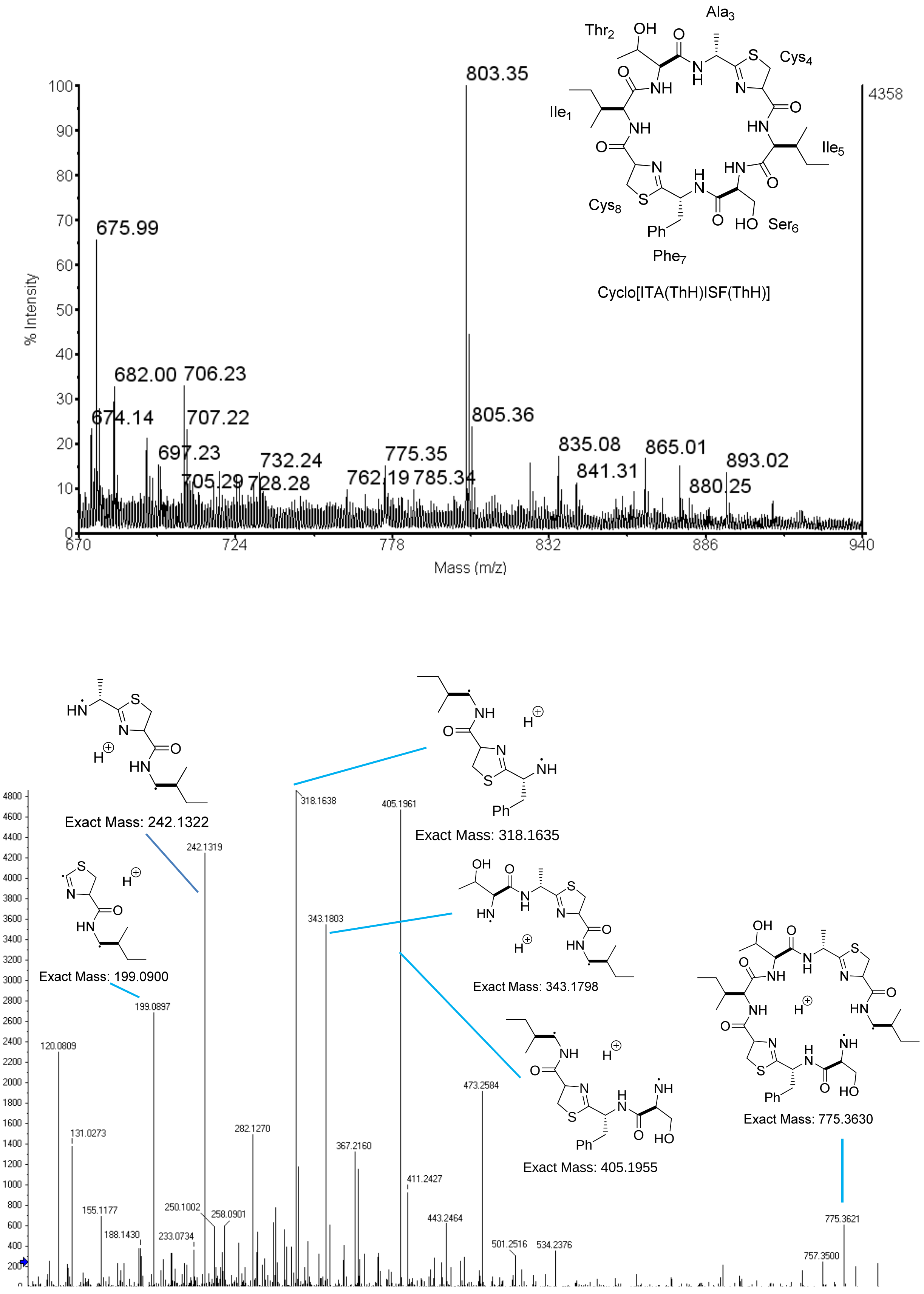


**Figure S30.** MALDI-MS and MS-MS data for compound **9**.

Compound **10**: Precursor peptide is Leader-GLEAS-K-GITACICVC-AYDGELE-Tag treated with TruD, trypsin and PatG$_{mac}$.

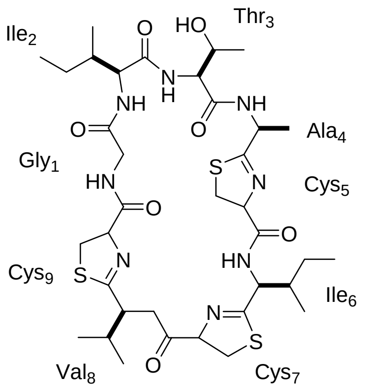


Cyclo[GITA(ThH)I(ThH)V(ThH)]

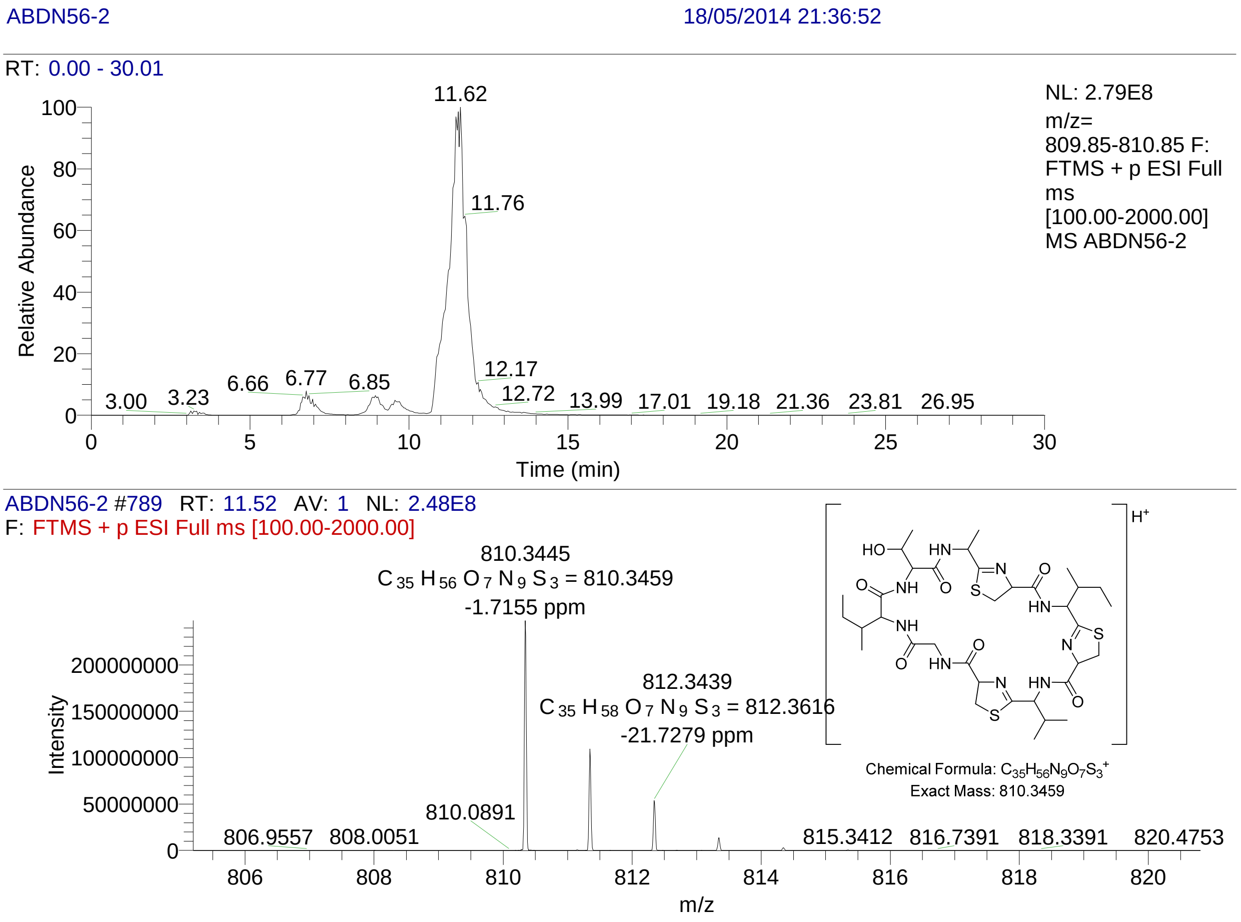


**Figure S31.** Accurate LC-MS data for compound **10**.

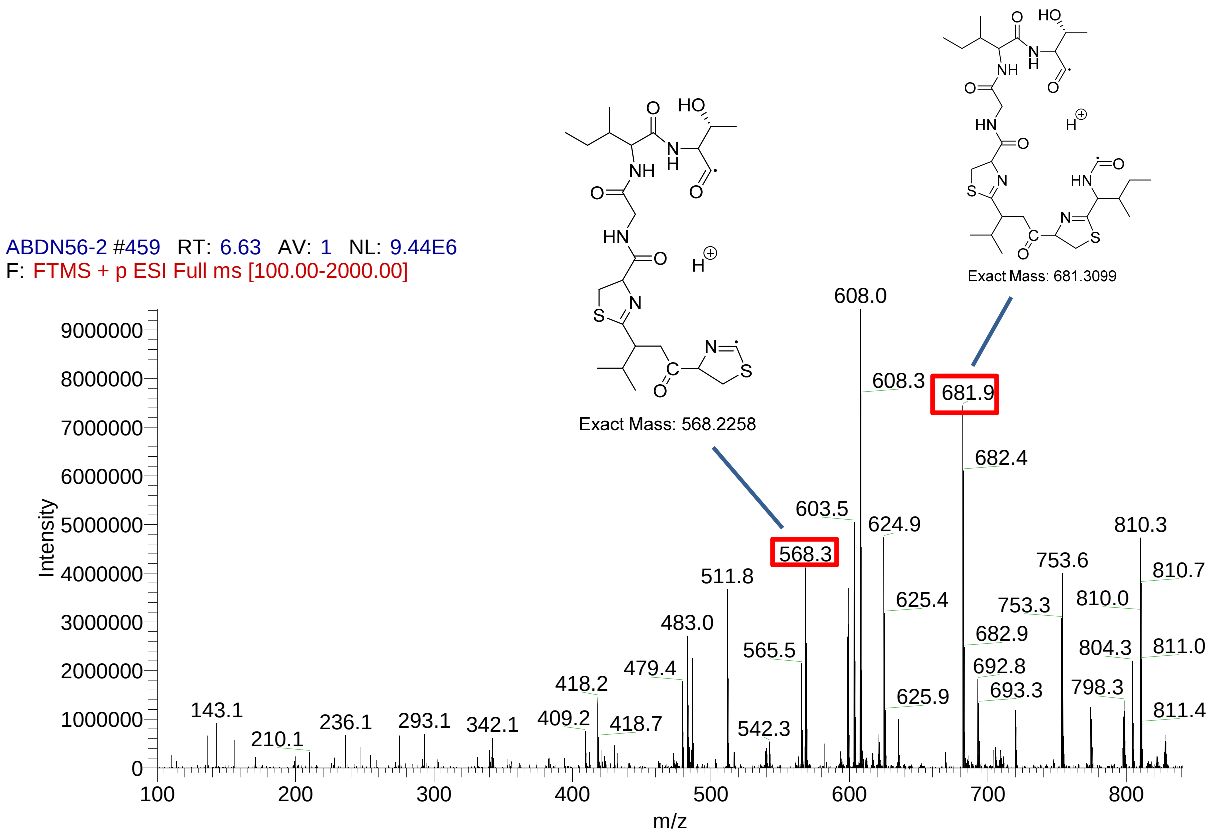


**Figure S32.** MS2 fragments for compound **10**.

<u>Compound 11</u>: Precursor peptide is Leader-GLEAS-K-<u>VCVCVC</u>-AYDGELE-Tag treated with TruD, trypsin and $PatG_{mac}$.

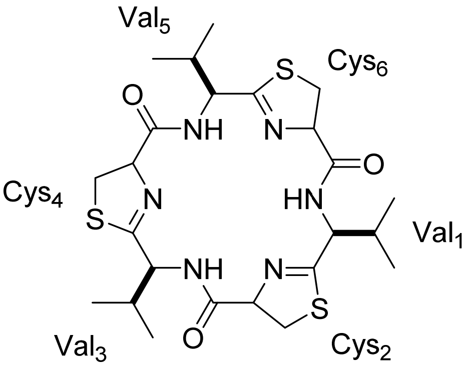


Cyclo[V(ThH)V(ThH)V(ThH)]

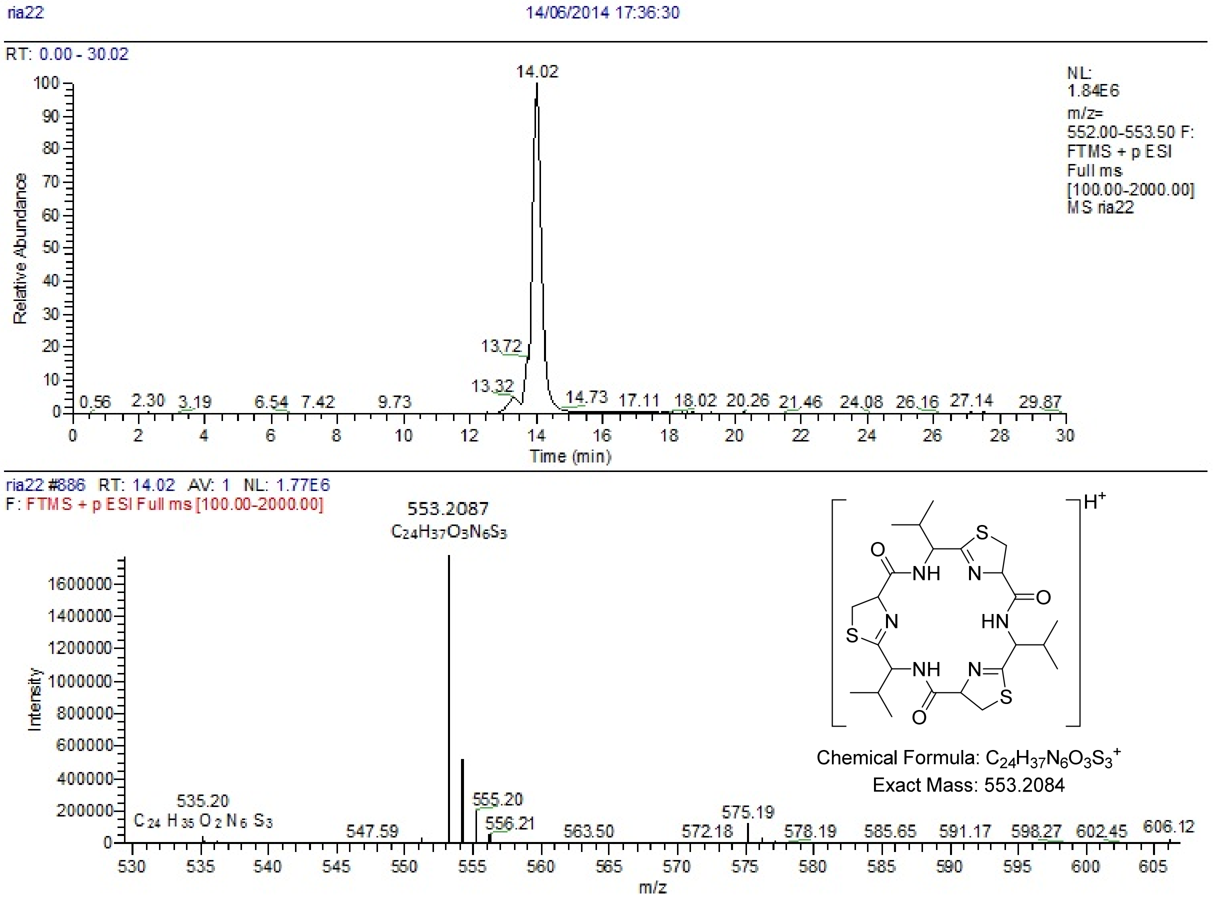


**Figure S33.** Accurate LC-MS data for compound **11**.

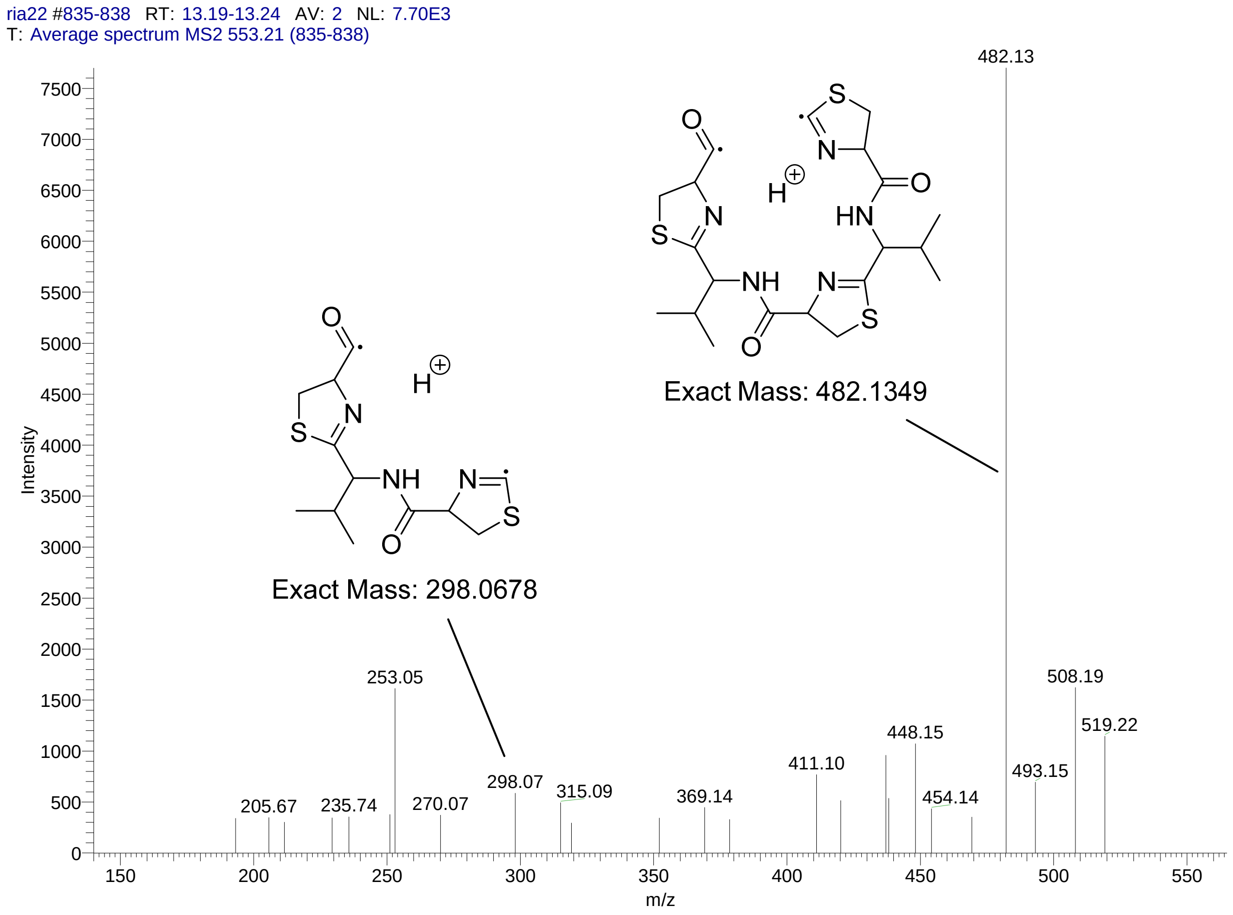


**Figure S34.** MS2 fragments for compound **11**.

Compound 12: Precursor peptide is Leader-GLEAS-K-ITMCITMC-AYDGELE-Tag treated with TruD, trypsin and $PatG_{mac}$.

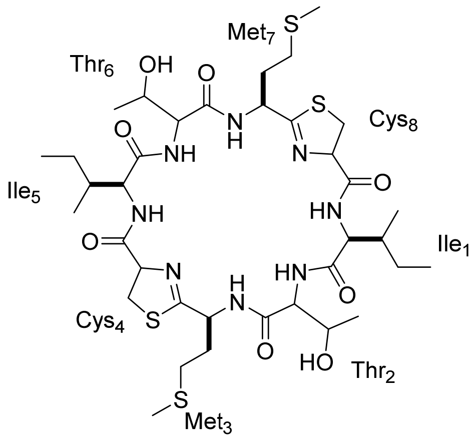


Cyclo[ITM(ThH)ITM(ThH)]

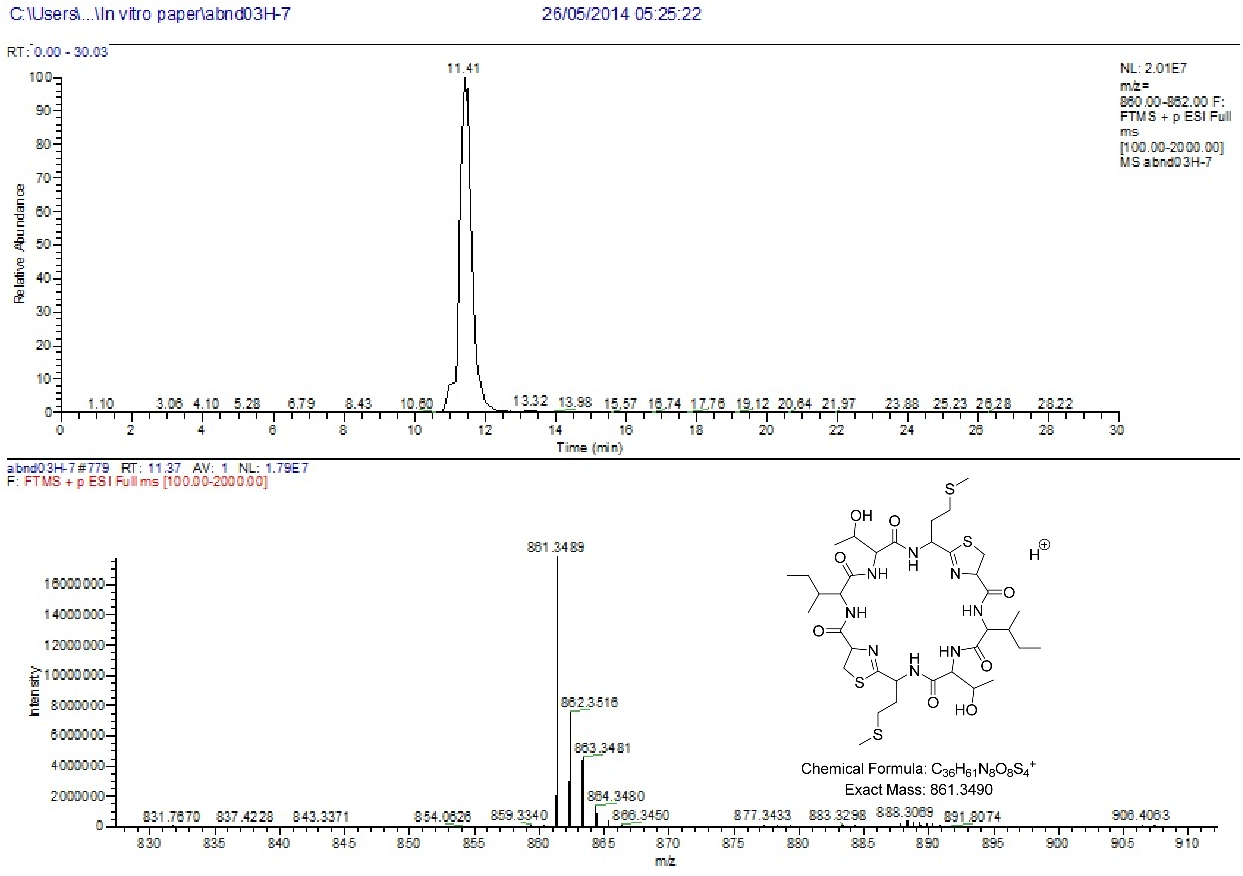


**Figure S35.** Accurate LC-MS data for compound **12**.

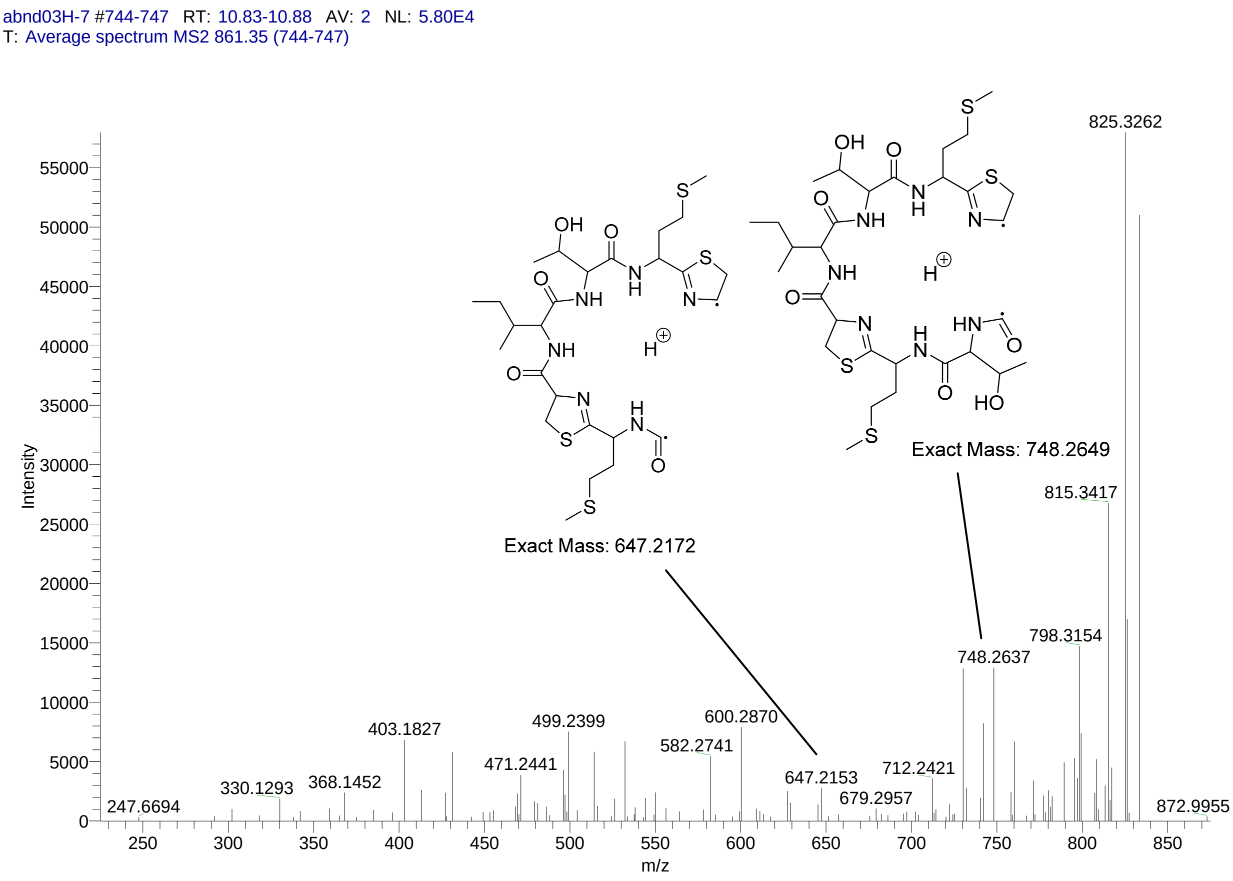


**Figure S36.** MS2 fragments for compound **12**.

Compound **13**: Precursor peptide is Leader-GLEAS-K-IFTVCICVC-AYDGELE-Tag treated with TruD, trypsin and $PatG_{mac}$.

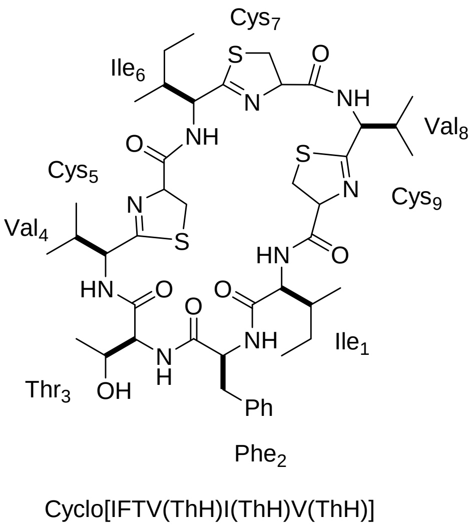


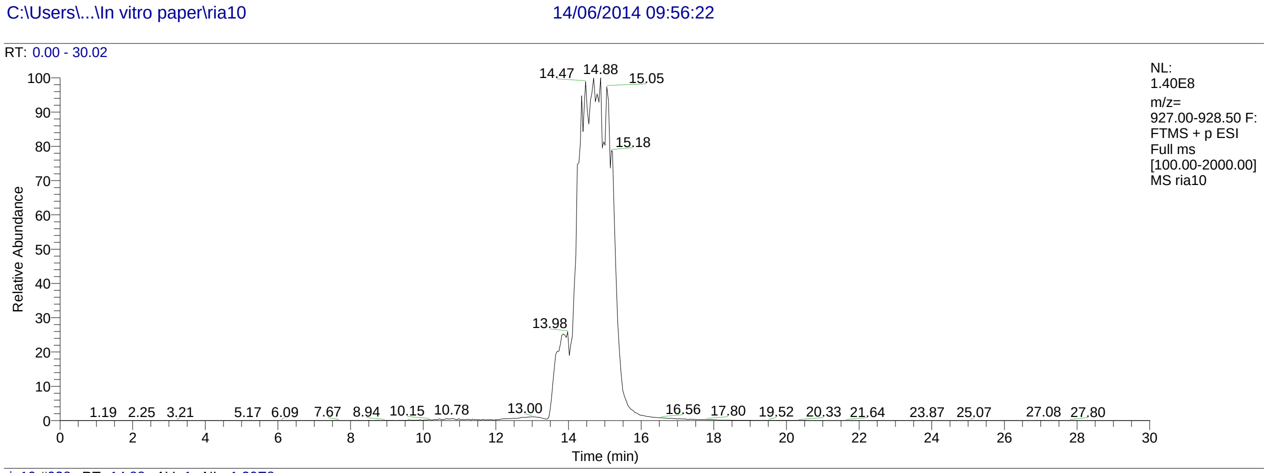


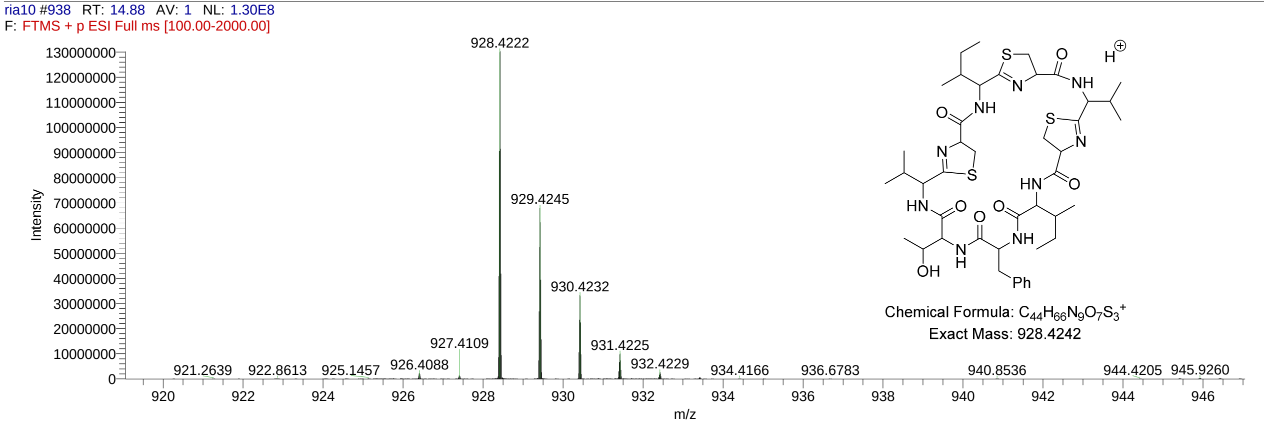


**Figure S37.** Accurate LC-MS data for compound **13**

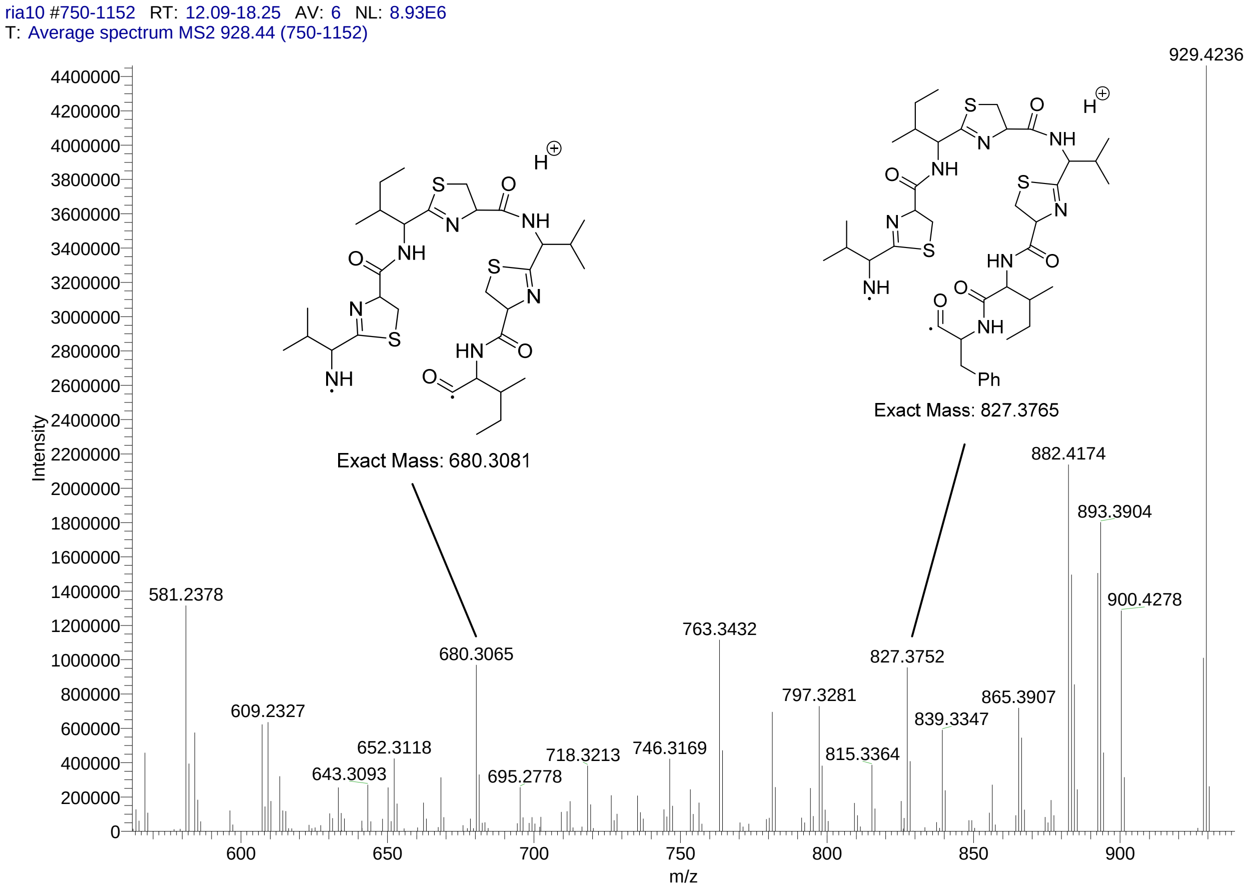


**Figure S38.** MS2 fragments for compound **13**.

Compound 14: Precursor peptide is Leader-GLEAS-K-ITACITYC-AYDGELE-Tag treated with TruD, trypsin and PatG$_{mac}$.

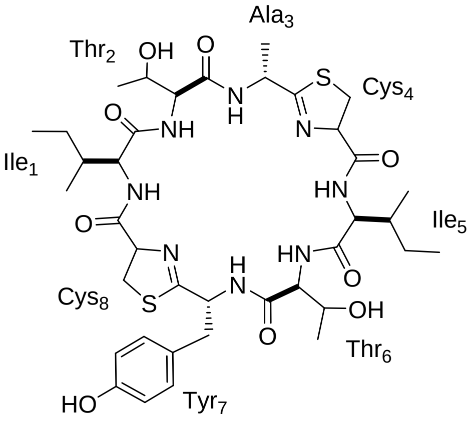


Cyclo[ITA(ThH)ITY(ThH)]

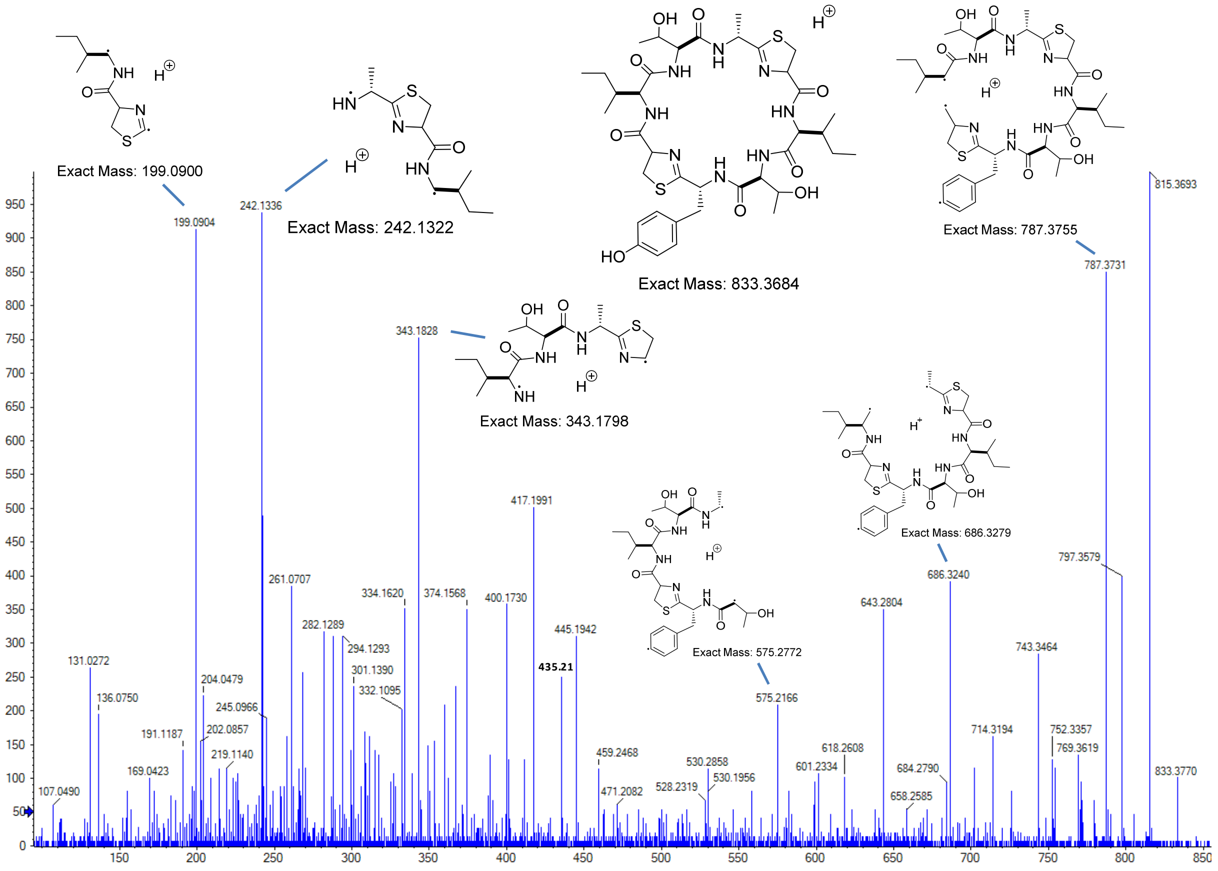


**Figure S39.** Parent ion and MS2 fragments for compound **14**.

Compound 15: Precursor peptide is Leader-GLEAS-K-ITACITYC-AYDGELE-Tag treated with PatD, trypsin and $PatG_{mac}$.

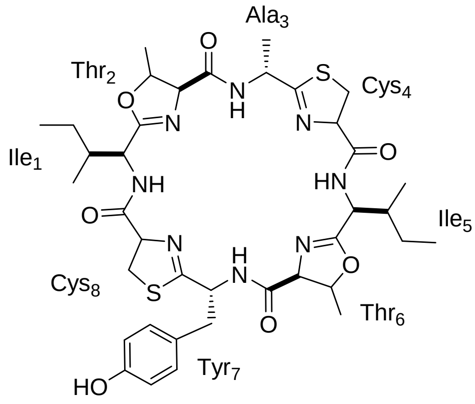


Cyclo[I(MeOxH)A(ThH)I(MeOxH)Y(ThH)]

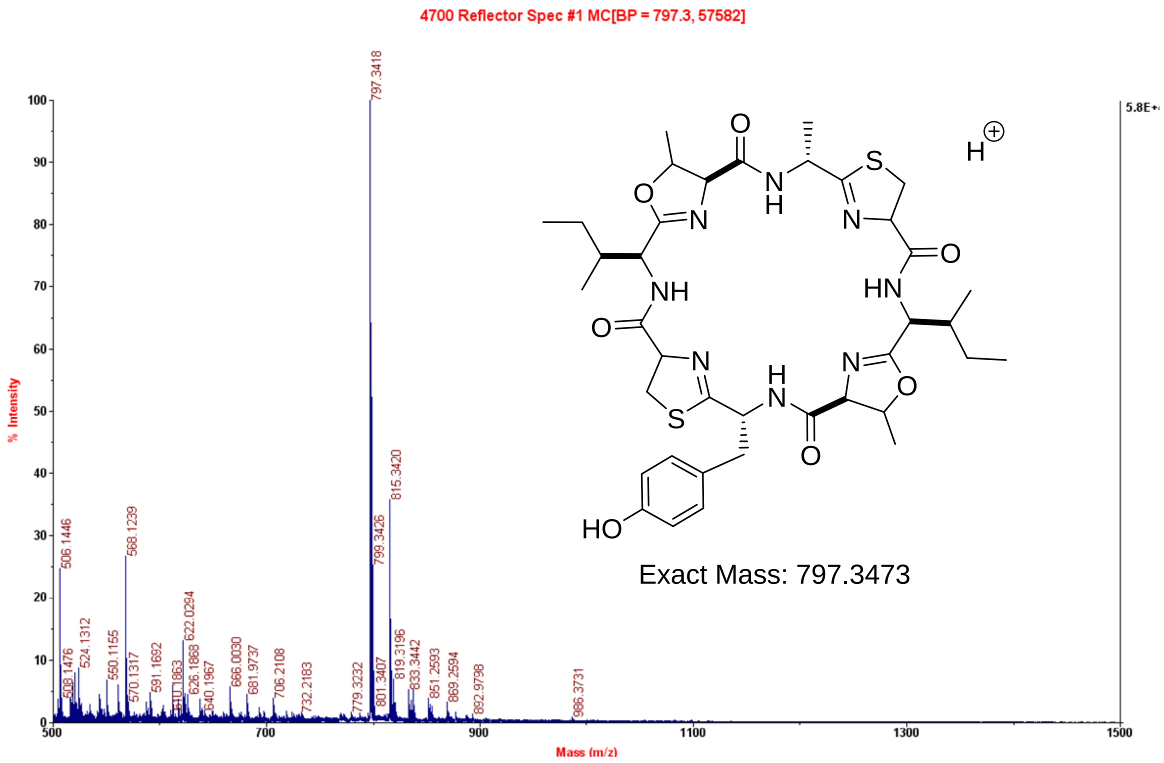


**Figure S40.** MS spectrum for compound **15**.

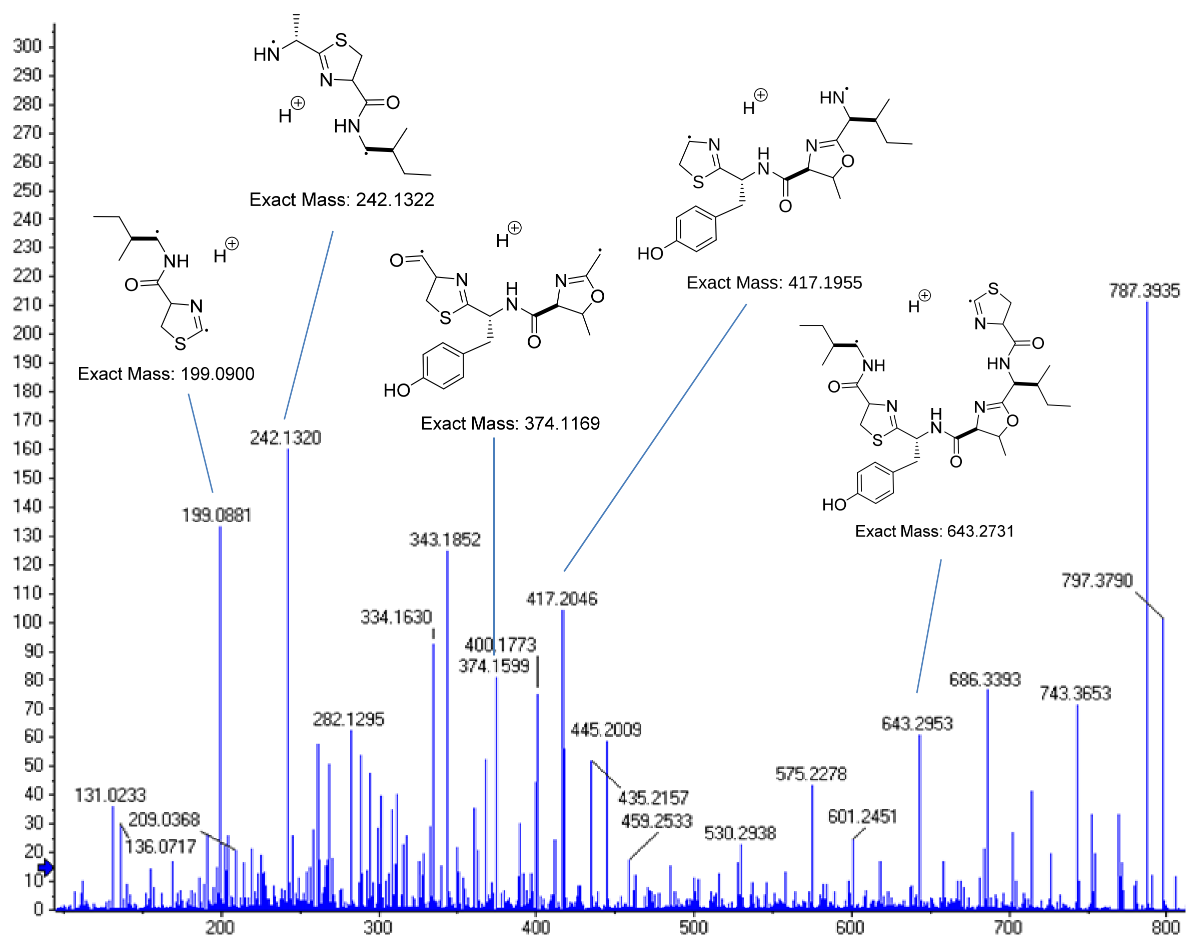


**Figure S41.** MS2 fragments for compound **15**.

<u>Compound **16**</u>: Precursor peptide is Leader-GLEAS-K- <u>IDACIDFC</u>-AYDGELE-Tag treated with TruD, trypsin and $PatG_{mac}$.

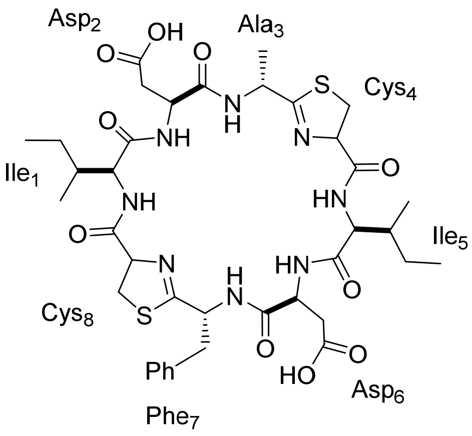


Cyclo[IDA(ThH)IDF(ThH)]

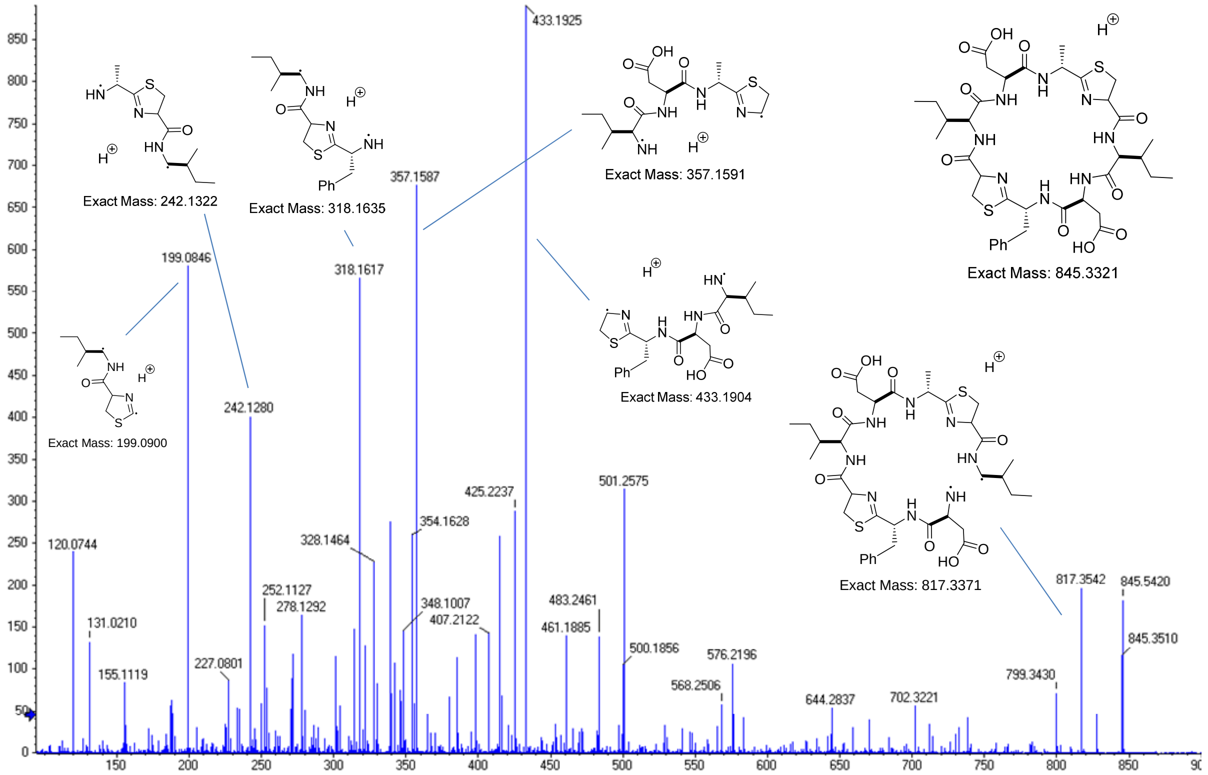


**Figure S42.** Parent ion and MS2 fragments for compound **16**.

Compound **17**: Precursor peptide is Leader-GLEAS-K-IACIMAC-AYDGELE-Tag treated with TruD, trypsin and $PatG_{mac}$.

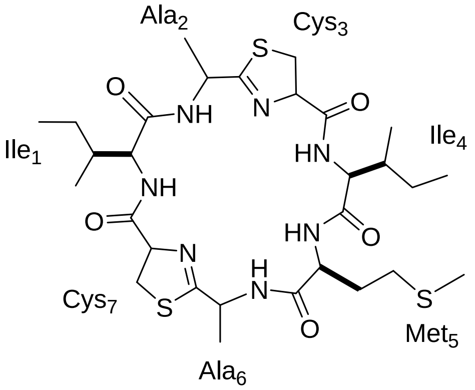


Cyclo[IA(ThH)IMA(ThH)]

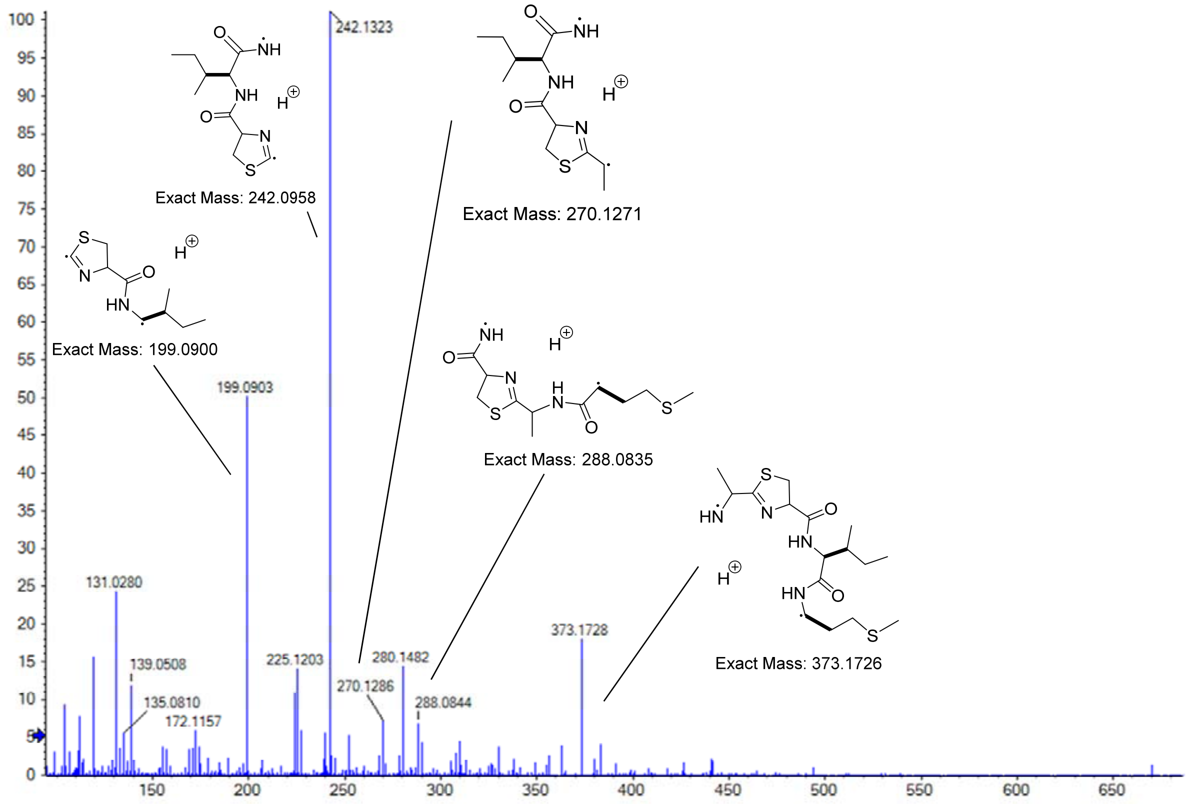


**Figure S43.** MS2 fragments for compound **17**.
